# Relationships Between the Microbial Composition and the Geochemistry and Mineralogy of the Cobalt-Bearing Legacy Mine Tailings in Northeastern Ontario

**DOI:** 10.3389/fmicb.2021.660190

**Published:** 2021-09-17

**Authors:** Brittaney Courchesne, Michael Schindler, Nadia C. S. Mykytczuk

**Affiliations:** ^1^School of the Environment, Laurentian University, Sudbury, ON, Canada; ^2^Department of Geological Sciences, University of Manitoba, Winnipeg, MB, Canada

**Keywords:** tailings, arsenates, bacteria, arsenic, cobalt, arsenic valence, neutral, biogeochemistry

## Abstract

Mine tailings host dynamic biogeochemical processes that can mobilize a range of elements from the host material and release them into the environment through acidic, neutral, or alkaline mine drainage. Here we use a combination of mineralogical, geochemical, and microbiological techniques that provide a better understanding of biogeochemical processes within the surficial layers of neutral cobalt and arsenic-rich tailings material at Cobalt, ON, Canada. Tailings material within 30-cm depth profiles from three tailings sites (sites A, B, and C) were characterized for their mineralogical, chemical and microbial community compositions. The tailings material at all sites contains (sulf)arsenides (safflorite, arsenopyrite), and arsenates (erythrite and annabergite). Site A contained a higher and lower amount of (sulf)arsenides and arsenates than site B, respectively. Contrary to site A and B, site C depicted a distinct zoning with (sulf)arsenides found in the deeper reduced zone, and arsenates occurring in the shallow oxidized zone. Variations in the abundance of Co+As+Sb+Zn (Co#), Fe (Fe#), total S (S#), and average valence of As indicated differences in the mineralogical composition of the tailings material. For example, material with a high Co#, lo Fe# and high average valence of As commonly have a higher proportion of secondary arsenate to primary (sulf)arsenide minerals. Microbial community profiling indicated that the Cobalt tailings are primarily composed of Actinobacteria and Proteobacteria, and known N, S, Fe, methane, and possible As-cycling bacteria. The tailings from sites B and C had a larger abundance of Fe and S-cycling bacteria (e.g., *Sulfurifustis* and *Thiobacillus*), which are more abundant at greater depths, whereas the tailings of site A had a higher proportion of potential As-cycling and -resistant genera (e.g., *Methylocystis* and *Sphingomonas*). A multi-variate statistical analysis showed that (1) distinct site-specific groupings occur for the Co # vs. Fe #, Co# vs. S#’s and for the microbial community structure and (2) microbial communities are statistically highly correlated to depth, S#, Fe#, pH and the average valence of As. The variation in As valence correlated well with the abundance of N, S, Fe, and methane-cycling bacteria. The results of this study provide insights into the complex interplay between minerals containing the critical element cobalt, arsenic, and microbial community structure in the Cobalt Mining Camp tailings.

## Introduction

Understanding the interactions between minerals and microorganisms is essential in the interdisciplinary and growing field of biogeochemistry, an integration of geology, chemistry and biology (Parkes, 1998; Canuel, 2007; Konhauser, 2007; Ehrlich and Newman, 2009; Gadd, 2010). Studying mineral-microbial relationships can enhance our knowledge of the biogeochemical cycling of elements, the formation of new and/or biogenic minerals, mineral dissolution, and chemical transformation of elements (Gadd, 2010). In mining operations, waste by-products including tailings, are host to complex microbial and geochemical processes. These processes can either sequester or mobilize a range of elements, which may result in their enrichment in the tailings material or their release into the environment through acidic, neutral, or alkaline mine drainage. At the Cobalt Mining Camp (CMC) in Northeastern Ontario, Canada, mine tailings contain elevated concentrations of cobalt (Co) and arsenic (As). Cobalt is widely used in alloy manufacture and aerospace materials (Kapusta, 2006), whereas As is a known toxicant causing adverse health effects on living organisms (Bissen and Frimmel, 2003). Cobalt is considered a critical element, as it is often extracted as by-product of other metals (Cu, Ni, and Ag), and its future supply depends on mining developments of the host metal and can be constrained due political instability, discouraging mining policies, or trade restrictions (European Commission, 2010).

An understanding of the biogeochemical cycling of both elements in the mine tailings at the CMC is therefore critical in terms of the environmental impact of the current mine tailings, the addition of mine waste during future mining operations or their potential bioleaching from the existing mine waste.

This study applied for the first-time a multi-variate statistical analyses to gain a critical understanding on the relationships between the abundance of Co-bearing (sulf)arsenides and arsenates, geochemical parameters such as pH and Eh, geomorphological parameters such as depth, and microbial community compositions in the near-neutral surficial tailings material at the CMC.

Numerous studies have characterized the relationship between microbial community structure and the variation of geochemical and mineralogical parameters in tailings material of an acidic pH (e.g., Bruneel et al., 2005; Diaby et al., 2007; Kock and Schippers, 2008; Wakelin et al., 2012). Studies, such as these, have most importantly (1) identified Fe- and S-cycling bacteria as a prominent metal-cycling bacterial community capable of the accelerated dissolution of Fe^3+^-bearing minerals (e.g., goethite); and (2) characterized the role of both biotic and abiotic processes involved in sulfide-mineral weathering.

The characterization of microbial communities and their relation to geochemical and mineralogical factors in As-rich mine tailings of near neutral-pH are less commonly studied even though As-cycling is known to play an important role on the bacterial community structure and composition (Guan et al., 2017). For example, within the As- and Sb-rich near-neutral mine tailings in Slovakia, Majzlan et al. (2011) showed that (a) bacteria commonly found in As-contaminated soils were dominant (i.e., aerobic heterotrophic bacteria of the *Sphingomonas*, *Caulobacter*, and *Janthinobacterium* genera), (b) siderite (FeCO_3_) formation was the result of Fe-reducing bacteria (IRB) such as *Geobacter* and *Rhodoferax* spp., and that (c) acidophilic microorganisms (e.g., *Acidithiobacillus* spp.) were able to thrive in predominantly neutral-pH tailings environments, specifically within microenvironments around altered and/or decomposing sulfide grains.

Additionally, Guan et al. (2017) showed that within As-rich soils and sediments contaminated by gold mining activities, the concentration of As was the most important factor influencing the microbial composition (e.g., *Clostridium* was the dominant bacterial genus within high As samples), with total organic carbon (TOC) and the concentrations of Fe and Mn having a less pronounced effect. The authors also showed that the bacterial communities developed an As-tolerance through phylum-level horizontal gene transfer, mainly within the Proteobacteria phylum.

No prior microbiological studies have been completed on the tailings material in the Cobalt Mining Camp. However, Little et al. (2020) identified As-tolerant diatoms such as *Achnanthidium minutissimum* and diatom populations that correlated to the abundance of As, during a paleolimnological and microbial study on several lakes within the CMC. The authors concluded that lake depth and pH were the significant drivers in community compositional changes and that the concentrations of As in the studied organisms had only minor effects on the compositions of the microbial communities.

Kelly et al. (2007); Kwong et al. (2007) both calculated the abundance of sulfate-reducing bacteria (SRB), IRB, and acid-producing bacteria (APB) in wetland sediments of the Cobalt mining camp using the most probable number (MPN) method. Within sediment samples from the Farr Creek drainage area, Kelly et al. (2007) identified (a) similar bacterial populations within the upper 50 cm, (b) the occurrence of similar abundances of SRB and IRB (therefore concluding that oxic and anoxic conditions existed throughout the wetland), and that (c) the abundance of SRB, IRB, and APB varied with depth. Furthermore, Kwong et al. (2007) analyzed sediment samples from the Farr and Mill Creek wetlands and determined that As co-precipitation as sulfides (such as framboidal pyrite) could be due to microbial sulfate reduction, and that the dissolution of As-bearing minerals, along with changes in redox conditions within surface layers, have resulted in As mobilization.

The speciation of As in natural waters and sediments around the CMC has also been studied. Beauchemin and Kwong (2007); Chen et al. (2019) characterized the speciation of As in lake sediments near Cobalt and Crosswise Lake, close to Mill Creek and in submerged tailings underneath the Farr and Mill Creek streams. Chen et al. (2019) showed that As^5+^ was the dominant As valence in the lakes and streams that were affected by the As-contaminated tailings, and that pH, temperature, light and the presence of nitrate and chloride strongly affected the stability of As^3+^ in these waters. Beauchemin and Kwong (2007) showed that (1) As primarily sorbed to Al-(hydr)oxides over Fe-(hydr)oxides (the latter of which occurred in trace amounts)*;* (2) As^5+^-species were reduced to more soluble As^3+^-species in the absence of soluble C, resulting in the competitive binding of arsenate and phosphate groups to adsorption site on Al-(hydr)oxides*;* and (3) that prolonged flooding periods, as well as the addition of sufficient soluble C, could stimulate the microbial reduction of As, favoring the stabilization of arsenide-bearing minerals.

Thus, this study continued our efforts to elaborate the metal-mobilizing microbial communities naturally present in the CMC tailings materials. In a previous study, we identified important mineralogical-geochemical relationships within two tailings profiles. We could specifically show that tailings material became depleted in Fe and enriched in Co and As with increasing degree of alteration of the original tailings material (enriched in (sulf-)arsenides). The trend was visualized in plots with the Co-As-Sb-Zn number (Co#) vs. the Fe number (Fe#), and were defined as:


(1) C⁢o-A⁢s-S⁢b-Z⁢n⁢n⁢u⁢m⁢b⁢e⁢r⁢(C⁢o⁢#)=(C⁢o+A⁢s+S⁢b+Z⁢nC⁢o+A⁢s+S⁢b+Z⁢n+A⁢l)⁢x⁢ 100%

and


(2) F⁢e⁢n⁢u⁢m⁢b⁢e⁢r⁢(F⁢e⁢#)=(F⁢eF⁢e+A⁢l)⁢x⁢ 100%.

The authors proposed that abiotic processes such as mineral replacement reactions and the complexation of Fe by carbonate species could have resulted in the mobilization and thus depletion in Fe with increasing degree of alteration. As the role of microbes during this process were not addressed by Courchesne et al. (2021), this study specifically investigated whether enrichments in Co and As, and depletion in Fe at certain depths within each tailings site are products of microbial activities, or whether the enrichments or depletions in Co, As, and Fe are an abiotic process.

For this purpose, we focused this study on characterizing the mineral-microbial relationships in the CMC tailings, and determined whether variations in the geochemistry, mineralogy and average valence of As affected the composition of the microbial communities. These objectives were accomplished using geochemical, mineralogical and molecular biology tools to better correlate difference in microbial community composition within the natural gradient of conditions found within the CMC tailings.

## Materials and Methods

### Study Area

**FIGURE 1:**
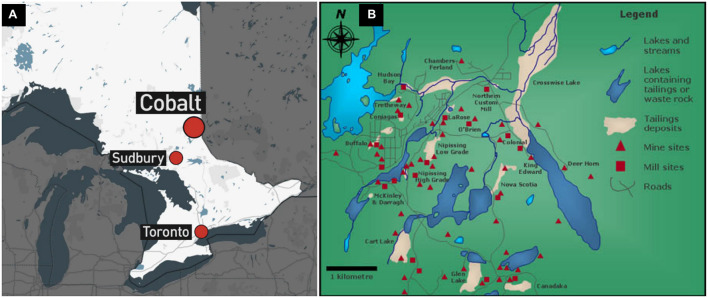
Maps indicating **(A)** the location of Cobalt in Ontario, relative to Sudbury and Toronto and **(B)** the mines, mills, and tailings deposits in the Cobalt Mining Camp.

The Cobalt Mining Camp (CMC) in Northeastern Ontario contains a total of 16 distinct tailings sites (Figure 1). Three tailings sites within the Farr Creek drainage basin were chosen on the basis of their previously reported As and Co content (Ontario Ministry of the Environment, 2011). These tailings sites are referred hereafter to as sites A, B, and C. Courchesne et al. (2021) describes in detail the historical background, geographical settings and the chemical and mineralogical compositions of the tailings sites A and B. Site A occupies an area around a lake within the town of Cobalt and its tailings material originated from five different mines. Site B is located immediately down-hill from a former processing mill at which its tailings material was derived (Dumaresq, 1993). Site C occurs in a low-lying wetland, with a majority of its tailings having been flushed into a nearby creek and Cobalt lake, due to erosion and/or dam failure. Its tailings material originated from a mill which processed high-grade ore using a cyanide-mercury amalgamation technique (Anderson, 1993).

### Sampling Methods and Storage

**FIGURE 2:**
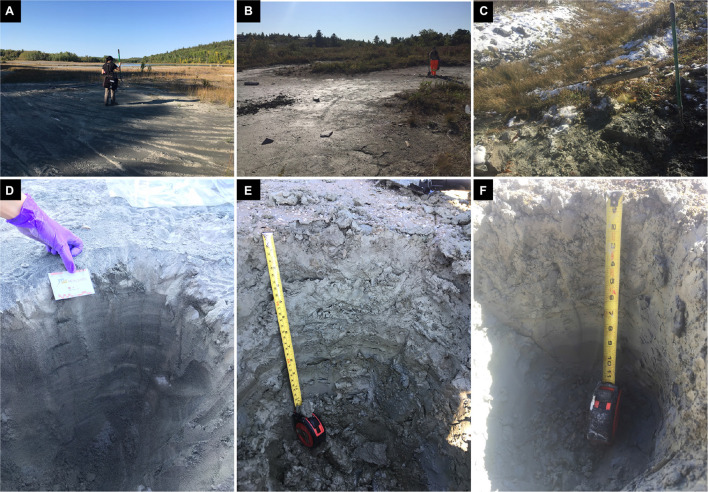
Photographs of the three tailings sites with their 30-cm depth profiles, showing site A **(A,D)**, B **(B,E)**, and C **(C,F)** at the day of sampling.

Three tailings profiles were sampled in September 2019 (sites A and B) and October 2019 (site C) (Figure 2). The profiles at sites A and B were sampled in close proximity to the sites sampled in our previous study from the same area (Courchesne et al., 2021). The profile at site C was taken directly beside a former tailings dam. Each of the three tailings profiles were sampled using a small hand shovel in 5-centimetre (cm) increments to a depth of 30 cm, outlined in Table 1, using nitrile gloves and ethanol wash to minimize cross-contamination. A total of 18 samples (∼1 L of material) were subsequently sealed in plastic bags, placed into a cooler with freezer packs in order to maintain a temperature of 4°C, and stored in a fridge at the Vale Living with Lakes Centre, Laurentian University. Shortly after arriving in the lab, approximately 100 g of each sample was placed into separate sterile plastic bags for DNA extractions and stored in a freezer at −20°C. All extractions were completed within a 3 month period following collection. A second subset was dried in an oven at 80°C for 2 days and then stored at room temperature until further analyses.

**TABLE 1:** The naming scheme for each sample, used in the taxonomic plots.

Sample ID	Site	Depth range (cm)
A-05	A	0–5
A-10	A	6–10
A-15	A	11–15
A-20	A	16–20
A-25	A	21–25
A-30	A	26–30
B-05	B	0–5
B-10	B	6–10
B-15	B	11–15
B-20	B	16–20
B-25	B	21–25
B-30	B	26–30
C-05	C	0–5
C-10	C	6–10
C-15	C	11–15
C-20	C	16–20
C-25	C	21–25
C-30	C	26–30

### Field Measurements

During sampling, contact pH and ORP measurements were taken using a handheld field instrument, YSI Quatro (YSI Environmental, OH, United States). A 4-point pH standardization was completed before sampling using standardized solutions of pH 2, 4, 7, and 10. ORP standardization was done using Zobell’s ORP solution (228 mV). Both pH and ORP were completed with a 1:2 tailings to deionized (DI) water ratio. The ORP mV value was then converted to an Eh value simply by adding 200 mV to the ORP voltage (YSI Environmental, 2005).

### Total Carbon and Sulfur Analysis

The 18 tailings samples were analyzed for total carbon (CO_2(T)_) and total sulfur (S_(T)_) at the Geoscience Laboratories, Sudbury, Ontario. The samples were analyzed with a LECO CS844 where approximately 0.2 g of soil was combusted in a stream of purified O_2_ gas and passed over a heated catalyst, oxidizing total S and C to SO_2_ and CO_2_, respectively. The latter two gasses were then detected by two non-dispersive infrared cells (Amirault and Burnham, 2013).

### Chemical Analysis*-*Inductively Coupled Plasma Mass Spectrometry

Total chemical analysis (55 element series) of all 18 samples was carried out at the Perdue Central Analytical Facility, Sudbury, Ontario following the Environmental Protection Agency (EPA) method 3052 (EPA, 1996). A single digest was obtained through mixing 0.5 g (±0.002) sample, 9 mL HNO_3_, 2 mL HCl, 3 mL HF, and 2 g of H_3_BO_3_ following the procedure of Wilson et al. (2006). The digests were diluted by factors of 1000 or 10000 and analyzed on a NexION© 1000 ICP-MS using a 7-point calibration curve. The internal standards Ru-101 and Re-185 were used for the corrections of drifts for the low to medium and heavy mass isotopes, respectively. For quality assurances, a method blank, duplicate and certified reference material (TILL-1; Lynch, 1990) was analyzed after every 9th sample.

### Powder X-ray Diffraction

Three samples from each depth profile were prepared for X-ray powder diffraction. These samples were chosen based on their Co, As, and Fe concentrations or their average valence of As. Nine X-ray diffraction patterns were recorded with either a Philips 159 PW 1729 or a Bruker D 5000 X-ray diffractometer using Cu-Kα radiation (λ = 1.5418 Å) at 40 kV and 30 mA. Diffraction patterns were collected over a range of 5–75° and 5–65° 2θ with a step size of 0.02° and a counting time of 2 s step^–1^. All minerals identified both previous to this study and within this study, as well as their chemical formula, are listed in Supplementary Material Appendix A. Additionally, all recorded X-ray diffraction patterns are listed in the Supplementary Figures B1-B3.

### Scanning Electron Microscopy

A total of 7 samples were prepared for Scanning Electron Microscopy (SEM), two from sites A and B, and three from site C. The samples were embedded into epoxy pucks and polished using MicroPolish Alumina powder on 8-inch Nylon PSA Buehler discs. Six of the carbon coated epoxy pucks were analyzed using a JEOL 6400 SEM operating at 20 kV, equipped with both backscattered (BSE) and secondary (SE) electron detectors and an Energy Dispersive X-ray Spectrometer (EDS). Additional SEM work was carried out on one sample from site C with a FEI Quanta 650 FEG field emission scanning electron microscope at the Manitoba Institute of Materials at the University of Manitoba.

### DNA Extraction and 16S rRNA Gene Sequencing

Genomic DNA from the total microbial community within each of the 18 samples were extracted from 0.25 g of tailings material using the Sox DNA Isolation Kit (Metagenom Bio Inc., Toronto, ON, Canada). A method blank extraction (DNA extraction column loaded with sterile water) was also completed to determine whether background contaminants were present. Manufacturer protocols were followed, with an additional 10-min heating step at 70°C, after the addition of Solution Sox 1, to enhance DNA yields. The extracted DNA was quantified using a Take3 spectrophotometry system on a Synergy HI microplate reader (BioTek, Winooski VT, United States). DNA yield and quality was then tested by polymerase chain reactions (PCR) using Phire Green Hot Start Master Mix and 27F and 1492R primers (10 umol) in sterile Milli-Q water. The following PCR protocol was used: initial denaturation at 98°C for 30 s, followed by 30 cycles with denaturation at 98°C for 5 s, annealing at 55°C for 5 s, elongation at 72°C for 25 s, and a final elongation step at 72°C for 7 min. Verification of the PCR products was accomplished by gel electrophoresis on a 1% agarose gel. Samples that were not deemed to be of high enough yield or did not amplify, were re-extracted from frozen samples.

The extracted DNA was sent for library preparation and 16S rRNA gene sequencing on the Illumina MiSeq platform at Metagenom Bio Inc. (Waterloo, ON, United States). Library preparation was completed following MetagenomBio Inc. protocols (Bartram et al., 2011) and the Illumina Miseq Regent Kit V3. Sequencing was completed for 2 × 300 bp of the V4 region of the 16S rRNA gene using primers, 515F (Parada et al., 2016) and 806R (Apprill et al., 2015). Obtained raw sequence data were trimmed using of the BBDuk tool^1^, to remove primers from the forward and reverse reads. Using the DADA2 pipeline (version 1.8), the trimmed reads were processed, using the default filtering and merging parameters, with the sample interference being conducted using the pseudo-pooling method (Callahan et al., 2016). To assign taxonomy, the Silva132 database was used (Quast et al., 2013). Post taxonomy assignment, a rarefaction curve graph of all samples was generated, using the vegan package (Oksanen et al., 2008; Supplementary Figure C1). Contaminants in the data were determined using the BiocManager package decontam (Davis et al., 2018), using both frequency and prevalence methods, in order to remove common contaminants and to remove identified organisms in the method blank. A table showing the raw sequence data information of all samples, including the total number of sequences, the percent of sequences that were filtered out, and the percent of sequences recovered, per sample, is provided in the Supplementary Table C1. All sequence data was deposited into the NCBI SRA archive under accession PRJNA700089.

### X-ray Absorption Near-Edge Spectroscopy-VESPERS

X-ray absorption near-edge spectroscopy (XANES) spectra were characterized at the Very Sensitive Element and Structural Probe Employing Radiation from a Synchrotron (VESPERS) beamline at the Canadian Light Source, Saskatchewan, CA, in order to determine the average valence of As in the collected tailings material. XANES scans were collected on powdered samples in fluorescence mode using a Canberra 13-element Ge detector. The energy position was calibrated to the gold L3 edge (E_0_ = 11919 eV) using the maximum first derivative of the XANES spectrum of a gold foil standard. Samples were mounted at a 45° angle to the X-ray beam and the detector was positioned at a 90° angle to the X-ray beam. Three different spots on each sample were selected and each spot was scanned three times. The scan parameters of the XANES spectra with respect to E_0_ were from 11667 to 12166 eV with a step-size of 10 eV, 0.3 eV or 0.1 k for the pre-, near-, or post-edge region (respectively) per step, with a dwell time of 2 to 2.5s per step. Arsenic standards for As^5+^, As^3+^, As^1–^, and As^3–^ were a Na-arsenate (Na_2_AsO_4_⋅7H_2_O), arsenic trioxide, arsenopyrite and gallium arsenide, respectively, and were scanned with a 1s dwell time (Supplementary Figure D1). An accurate edge position for As could not be unequivocally determined in the X-ray absorption spectra of the samples from site A as they had a very low signal to noise ratio. A summary of the results from the XANES analysis can be found in the Supplementary Figures D2, D3.

### Statistical Analyses

Data and multivariate statistical analyses were conducted using R Core Team (2018), dplyr (Wickham et al., 2018), phyloseq (McMurdie and Holmes, 2013), and vegan packages. Graphs were generated using ggplot2 (Wickham, 2016).

Non-metric multidimensional scaling (NMDS) was implemented using the environmental fit function of the vegan package in R software (version 1.3.959). The relative abundance of microbial data (ASVs) was log transformed and subjected to the Bray-Curtis dissimilarity calculation, using the betapart package in R software. Each point of an NMDS plot represents the microbial community composition of a sample within a reduced 3D multidimensional space, with the distance between two points (i.e., two samples) representing the difference between the two samples microbial community composition. Hence, the closer two points are together, the more similar is there microbial community composition (the opposite is also true).

An Analysis of Similarities (ANOSIM) is a statistical test that was conducted using the anosim function in the vegan package in R software. The use of the ANOSIM here was to identify whether similarities (or differences) exist between the tailings sites. An ANOSIM provides an *R*-value associated with the similarities between the tested groups (Clarke, 1993). An *R*-value closer to 1.0 suggests that there are dissimilarities between groupings, while an *R*-value closer to 0.0 suggests dissimilarities within groups (Clarke, 1993). The ANOSIM results can be found in the Supplementary Figure E1.

## Results

The geochemistry and mineralogy of the tailings material at sites A and B were very similar to those studied by Courchesne et al. (2021) and, therefore, will only be briefly addressed. Additional information of the mineralogical and chemical composition of the tailings materials at the sites A, B, and C is given in the Supplementary Figures B,D,F,G.

**FIGURE 3:**
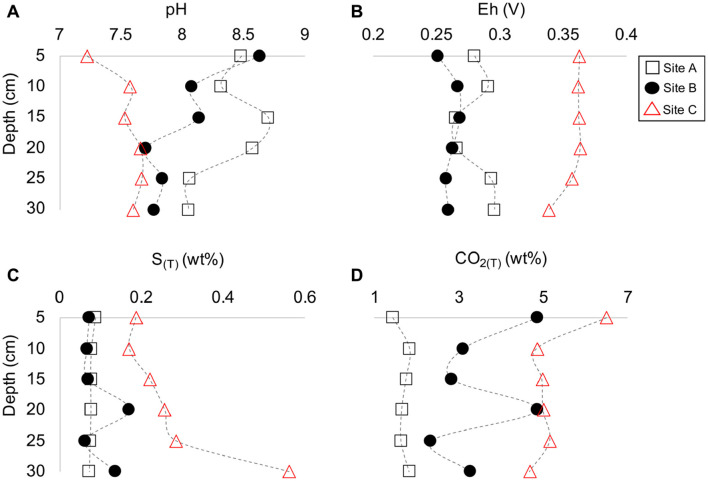
Depth profile plots of sites A to C indicating the change in pH **(A)**, Eh **(B)**, total sulfur **(C)**, and total CO_2_ **(D)** vs. depth (cm).

The tailings material at sites A and B depicted a gray-color tone (Figures 2D,E), whereas the material at site C had a light brown-color tone (Figure 2F). The occurrence of dark and/or light-colored laminations occurred in all three tailings profiles with reddish-brown oxidized material at depth (*D*) = 25 cm at site B and at *D* = 11 cm at site C, and a thick dark-colouration at *D* = 25 to 30 at site C. The average contact pH and Eh values for the materials were as follows at site A [8.4 (±0.2) and 0.28 V (±0.01)], site B [8.0 (±0.3), and 0.26 V (±5.7E–03]), and site C [7.6 (±0.2) and 0.36 V (±8.87E–03)] (Figures 3A,B). However, pH and Eh varied with depth for each profile. For example, material at *D* = 15 and 20 cm at site A had a significantly higher contact pH and lower Eh than those at other depths within the profile. Similarly, material at *D* = 20 cm at site B also had a much lower contact pH than the material above or below.

The tailings material at sites A and B had similar amounts of sulfur (S_(T)_) whereas the material at site C had higher concentrations of S_(T)_ (Figure 3C). The total carbon (C_(T)_) content in the tailings material differed significantly between the sites with the lowest and highest content at sites A and C, respectively (Figure 3D). The concentrations of S_(T)_ and C_(T)_ varied, similar to Eh and contact pH, with depth. For example, material with the lowest contact pH at site B (*D* = 20 cm) also had the highest amount of S_(T)_ and C_(T)_ (Figure 3). The highest amount of S_(T)_ for all characterized tailings material occurred at *D* = 30 cm at site C with 0.56 wt% S_(T)_ (Figure 3C).

### Changes in the Bulk Chemical Composition With Depth

**FIGURE 4:**
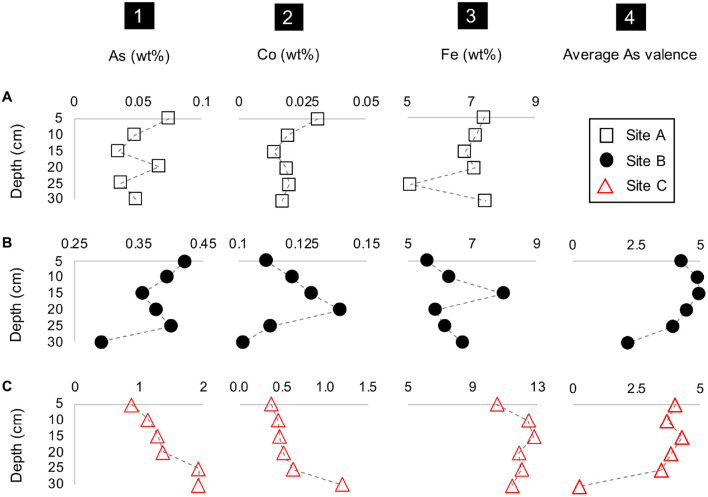
Depth profile plots of sites A to C (rows **A–C**) with the concentrations of (1) As, (2) Co and (3) Fe, and (4) the average As valence vs. depth (cm).

The concentrations of metal(loid)s of interest such as Co, As, Fe and the average valence of As also varied with depth (Figure 4). A common feature at all three sites was the occurrence of tailings material which was depleted in Fe and/or enriched in As and Co (at *D* = 25 cm at site A, at *D* = 20 cm at site B, and *D* = 30 cm at site C) (Figure 4). This depletion in Fe in the materials from sites B and C additionally coincided with a higher average As valence (Figure 4). Tailings material enriched in Fe also occurred at all three sites and coincides here with elevated concentrations of Co and As (*D* = 5 and 20 cm at site A, *D* = 15 cm at site B, and *D* = 15 cm at site C) (Figure 4). The material at site B depicted a higher average valence of As (+4.30) than site C with an As valence of +3.28 (Figure 4). A notable feature is the decrease in the average valence of As with depth at site C resulting in the lowest observed average valence of As with +0.25 at *D* = 30 cm (Figure 4). The percent range of As^5+^ in materials from site B is 43-88% and 12-73% in materials from site C (Supplementary Table F2).

Similar to the Co-Zn-Sb-As number (Co#) (Eq. 2) and Fe number (Fe#) (Eq. 3) (Courchesne et al., 2021), we define here the S number (S#) (Eq. 4) as:


(3) S⁢n⁢u⁢m⁢b⁢e⁢r⁢(S⁢#)=SA⁢l+S⁢x⁢ 100%

**FIGURE 5:**
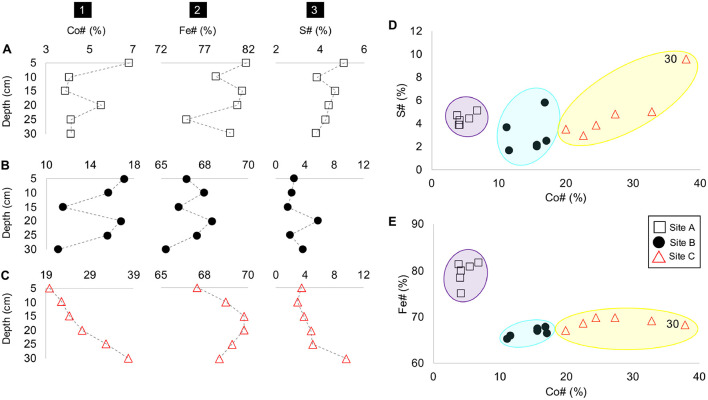
Depth profiles of sites A to C (rows **A–C**) with columns as (1) Co#, (2), Fe# and (3) S# vs. depth (cm); Co# vs. S# (in panel **D**); and Fe# (in panel **E**), with site groupings encircled in purple (site A), blue (site B), and yellow (site C).

Although, Co#, Fe#, and S# number vary with depth at all sites A, B, and C (Figures 5A-C), the tailings material at each site depicted a unique range of these numbers, especially with respect to the Co# and Fe#. For example, the tailings material at site A is characterized by the smallest and highest Co# and Fe# (Figures 5D,E). The tailings material at site B and C have similar Fe# with the material at site C depicting higher Co# and a much higher S# at *D* = 30 cm.

#### Arsenate and (Sulf-)Arsenides in the Tailings Material

Material from *D* = 15 to 25 cm at site A was chosen for SEM characterization as we were interested in mineralogical changes between material of high and low Fe# (Figure 5). At site B, tailings material at *D* = 10 and 25 cm were selected in order to examine the mineralogical composition of samples with similar Fe# and Co# (Figure 5). Tailings material from *D* = 20 to 30 cm at site C was chosen in order to characterize changes in the mineralogical composition with an increase in Co# and As valence and decreased in Fe# and S# (Figure 5).

**FIGURE 6:**
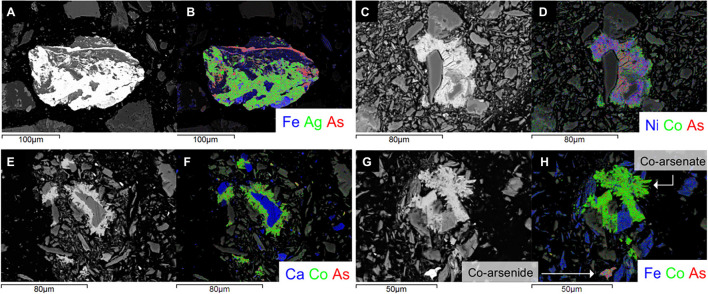
Scanning Electron Microscopy photomicrographs and EDS-chemical maps for representative grains from each tailings site. A silver-sulfide from site A at *D* = 15 cm **(A,B)** Ni, Co-arsenate coating on silicates from site B at *D* = 10 cm **(C,D)**; Co-As-bearing grains from site C at *D* = 20 cm **(E-H)**: Ni, Co-arsenate coating on carbonates **(E,F)**, and a Co-arsenate coating on silicates and an Fe, Co-arsenide **(G,H)** (indicated with an arrow and labeled accordingly).

In accord with previous observations by Courchesne et al. (2021) (sulf-)arsenides consist primarily of arsenopyrite, with minor amounts of chalcopyrite, safflorite, and cobaltite ± skutterudite, cobaltoan arsenopyrite, roselite, cobaltoan olivenite, argentobismutite and argentopyrite (based on the stoichiometry of the elements; Figure 6). Secondary arsenate minerals occured predominantly in mineral surface coatings on silicates and carbonates as Co-Ni-Zn-Fe-arsenates. Additionally, within samples of sites A and C, there are the minor occurrences of Fe (±Ti)-(hydr)oxides, that were absent in site B.

At site A (sulf-)arsenides were the dominant As-bearing phase at *D* = 15 cm, whereas the proportion of arsenates to arsenides was approximately equal at *D* = 25 cm (on the basis of 25 observed As-bearing mineral grains and surface coatings). At site B, arsenates were the dominant As phase over (sulf-)arsenides at both depths (on the basis of 39 As-bearing mineral grains and surface coatings) (Figure 6).

At *D* = 20 and 25 cm at site C, arsenates were also predominant over (sulf-)arsenides, but the ratio of arsenates to (sulf-)arsenides is smaller than at site B (on the basis of 69 As-bearing mineral grains and surface coatings) (Figure 6). Material at *D* = 30 cm of site C contained three distinct groups of: (1) sulf-arsenides; (2) arsenides; and (3) sulfides (Supplementary Figure G1). The former two groups were a mixture of Co-Fe-Ni-Zn rich arsenides and sulfarsenides, and corresponds to 33 of 47 examined grains, with the remainder and latter sulfide group consisting of Fe-sulfides and minor Zn-sulfides. Although Fe-sulfide grains can be slightly altered, none of the observed (sulf-)arsenide grains depicted visible (micrometer-size) mineral surface coatings (Supplementary Figure G1) or textures and crystal forms which would point toward their biogenic origin (e.g., framboidal textures).

### Geochemical vs. Mineralogical Trends in the Tailings Material

Although the sampling in this study was at a lower resolution than the study by Courchesne et al. (2021) (samples from every 5 cm vs. every 1 cm), the tailings profiles recorded at the sites A and B showed similar chemical and mineralogical features and trends:

(1) Tailings material at site B had a higher Co#, lower concentrations of Fe and higher proportions of secondary arsenate phases in the form of mineral surface coatings than the material at site A (Figures 5, 6);
(2) Tailings material at all sites had only minor amounts of Fe-(hydr)oxides;
(3) At sites A, B and C, tailings material with higher Co#’s had lower amounts of Fe than those with lower Co#’s (Figure 5); and
(4) At sites B and C, material with a high S# generally coincide with a lower As valence (e.g., at greater depths in sites B and C) (Figures 4, 5).

The tailings material at site C, which was not characterized by Courchesne et al. (2021), showed different geochemical and mineralogical trends, as well as much higher concentrations of Fe, As, S, Co and other metals. The tailings material at sites B and C did, however, follow a similar trend with respect to the average valence of As. Tailings materials at surface depths at both sites were characterized by higher valences of As (approaching +5.0), whereas material at greater depths (*D* = 30 cm) had lower average valences of As with +2.2 and +0.25, at sites B and C, respectively (Figure 4). The lowest measured valence of As in the bottom layer of site C coincided with a high number of Fe-sulfides and Co-Zn-Ni-Fe (sulf-)arsenides, and the absence of any arsenate-bearing mineral surface coatings (Supplementary Figure G1). Hence, a high Co# at the bottom of site C did not correspond to a high ratio of arsenates vs. (sulf-)arsenides (as observed at site A and B) as the latter minerals were minor rather than trace phases in the corresponding tailings material. Furthermore, the material at site C exhibited zonations similar to those observed in acidic tailings depth profiles, i.e., there was a reduced zone at greater depth, a transition zone and a surficial oxidized zone (Lottermoser, 2007). This zonation occurred between *D* = 30 and 25 cm and can be recognized by a gradual decrease in Eh, CO_2(T)_ content, Co#, S# and concentrations of As and Co, a decrease in the average As valence, and an increase in pH, Fe# and S_(T)_, i.e., there was a gradual oxidation of (sulf-) arsenides, leaching of metal(loid)s and (slight) increasing acidity toward the surface.

### Tailings Microbial Community Composition

In order to better characterize trends in the microbial data, taxa with a relative abundance below 2% (or 0.5% for Species-level description) were grouped together into a single category, and only taxa with a relative abundance >2% are described here. Additional taxonomic abundance graphs (Kingdom, Class, Family, and Species) can be found in the Supplementary Material Appendix H.

**FIGURE 7:**
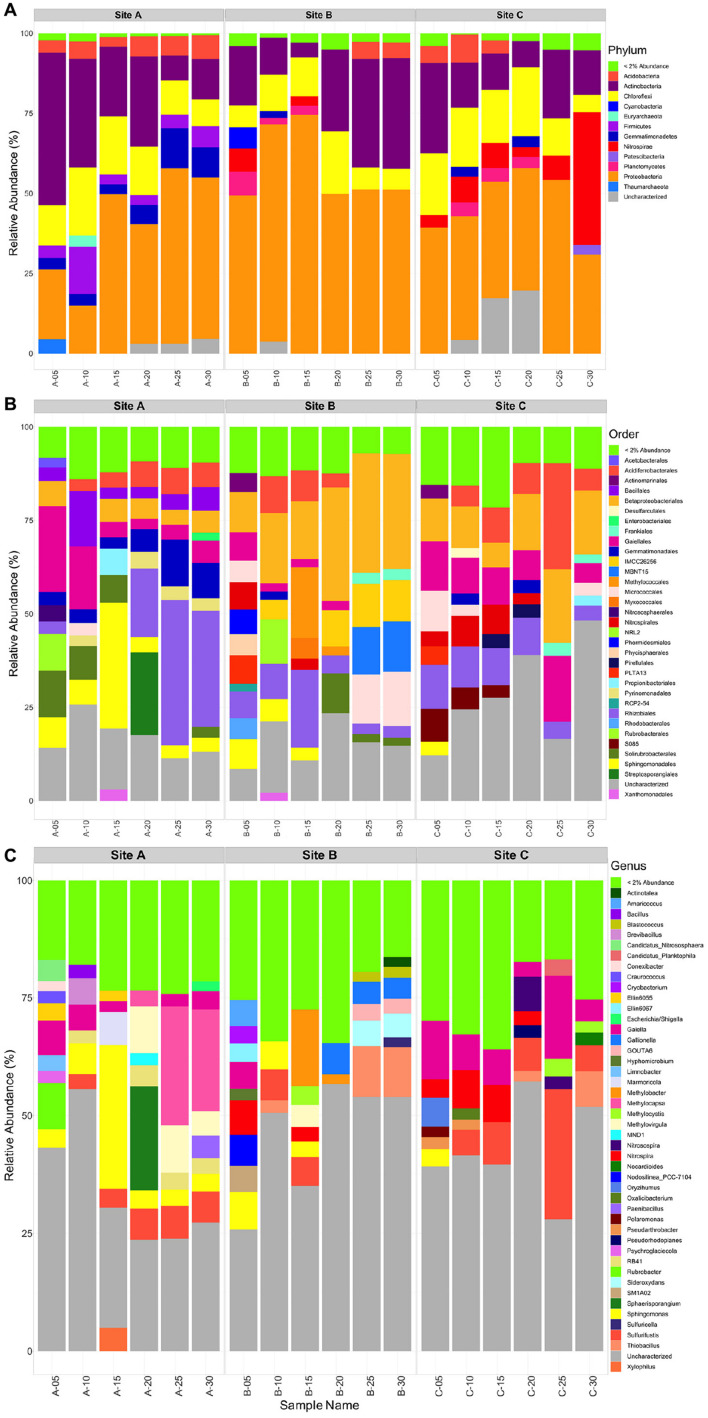
Taxonomic bar plot of the sites A, B, and C tailings microbial communities at the Phylum level **(A)**, Order level **(B)**, and the Genus level **(C)**.

Within materials from all three tailings sites, the bulk microbial community composition consisted mainly of the Proteobacteria (14-75%) and Actinobacteria (5-48%) phyla, with lesser amounts of Chloroflexi (5-21%), and Acidobacteria (3-9%) (Figure 7A). Additionally, tailings material at site B contained more Proteobacteria (49-75%) than materials from sites A (14-50%) and C (38-54%). A few minor differences existed between each tailings site: (1) tailings material at site A depicted the only occurrence of Firmicutes (4-13%) and had a comparatively higher relative abundance of Gemmatimonadetes (3-12%) and (2) there were less Actinobacteria (8-28%) and a higher abundance of Nitrospirae (41% at *D* = 30 cm) in the material at site C relative to those at sites A and B.

At site A, the community was largely composed of Acidoferrobacterales, Bacilalles, Betaproteobacteriales, Gaiellales, Gemmatimonadales, Pyrinomodales, Rubrobacterales, Rhizobiales, Solirubrobacterales, and Sphingomonadales (Figure 7B). At *D* = 0, 5, and 15 cm an abundance of Gaiellales (5-25%), Solirubrobacterales (7-13%), and Sphingomonadales (6-31%) occurred. At *D* = 20, 25, and 30 cm a greater abundance of Gemmatimonadales (6-14%), and Rhizobiales (18-40%) was present. The Bacillales, Betaproteobacteriales and Acidoferrobacterales were consistent throughout site A ranging from 3 to 12%. Corresponding genera that could be identified from the above Orders included: *Sphingomonas* present throughout the tailings profile at site A and up to 30% at *D* = 15cm, *Gaiella* (2-7%), *Nocardiodes* (20% at *D* = 20), *Rubrobacter* (10% at *D* = 0 cm), *Sulfurifustis* (3-7.5%), *Methylocapsa* (3-25%), and *Methylovirgula* (5-10%), with the latter three being more abundant in the lower half of the tailings profile (*D* = 20 to 30 cm) (Figure 7C).

At site B the community was largely composed of Acidoferrobacterales, Betaproteobacteriales, Gaiellales, Frankiales, Nitrospirales, Methylococcales, Micrococcales, Myxococcales, MBNT15, Rubrobacterales, Rhizobiales, Solirubrobacterales, and Sphingomonadales (Figure 7B). The surface community was diverse with no order comprising > 10%, whereas at all other depths Betaproteobacteriales (12–33%), Methylococcales (13% at *D* = 20 cm), Rhizobiales (2–20%), and Micrococcales (6–12%) dominated. Corresponding genera that could be identified from the above Orders included: *Gallionella* (3–5% at *D* = 20–30 cm), *GOUTA6, Gaiella* (4%), *Nitrospira* (2–6%), *Hyphomicrobium* (2.5%), *Methylobacter* (2–16%), *Methylocystis* (4%) and *Methylovirgula* (5%), *Sideroxydans* (6%), *Sphingomonas* (3–6% in *D* = 0–15 cm), *Sulfurifustis* (5%), and *Thiobacillus* (9% at *D* = 25 and 30 cm) (Figure 7C).

At site C, the community was largely composed of Acidoferrobacterales, Betaproteobacteriales, Frankiales, Gaiellales, Gemmatimonadales, Nitrospirales, Micrococcales, Pirellulales, Rhizobiales, and SO85 (Figure 7B). At the surface *D* = 0–15 cm, Acidoferrobacterales (3–6%), Betaproteobacteriales (3–10%), Gaiellales (7-12%), Micrococcales (2–7%), Rhizobiales (7–12%), and S085 (3–5%) dominated. At *D* = 20–30 cm, Acidoferrobacterales (3-25%), Betaproteobacteriales (13–18%) increased in abundance with similar amounts of Gaiellales (4–16%) and Rhizobiales (3–7%) as in the surficial layers. Notably, the class Thermodesulfovibrionia (uncharacterized Order) occurred in tailings at site C, at *D* = 25 and 30 cm with a relative abundance of 5 and 41% (Supplementary Figure H2). Corresponding genera that could be identified from the above Orders included: *Gaiella* throughout the tailings profile (3–18%), *Nitrospira* within the top 20 cm’s (3–8%), *Sulfurifustis* (6–28%) and *Thiobacillus* (2–7.5%) at *D* = 25 and 30 cm, respectively, and *Nitrosospira* at *D* = 20 and 25 cm (∼3 and 7%) (Figure 7C).

### Multivariate Analyses of Microbial Community Composition and Geochemistry

#### Analysis of Similarities

An analysis of similarities (ANOSIM) test was used to evaluate the relationships between the microbial community compositions, the environmental (or field parameters), and geochemical variables between each site, the data for which can be found in Figure 1 of Appendix E. The ANOSIM test resulted in an *R*-value of 0.742 with a *p*-Value of 0.001. An *R*-value closer to 1 implied a high dissimilarity between tested groupings, whereas a *p*-Value < 0.05 implied a high statistical significance. This indicated a strong, statistically significant difference in the microbial communities based on the site grouping (data for this test can be found in Supplementary Table F1).

#### Non-metric Multidimensional Scaling Analysis

**FIGURE 8:**
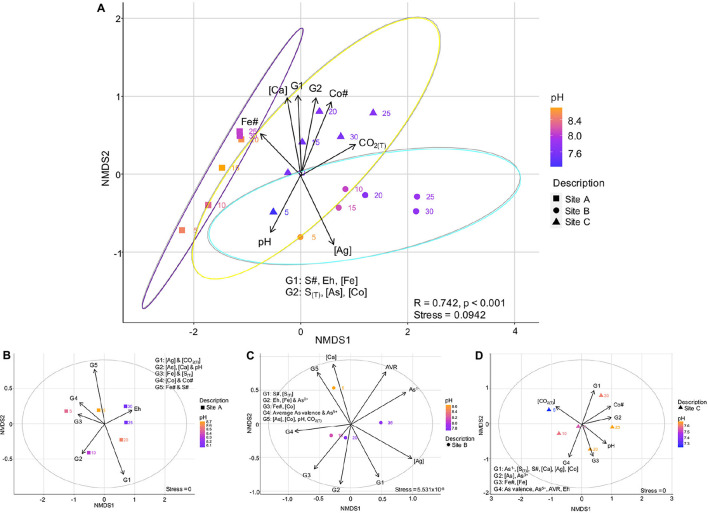
Non-metric multidimensional scaling plot showing the variation in environmental and geochemical variables with site, and their effect on the microbial communities of sites A, B, and C **(A)**; Site-specific NMDS plots showing how the enviromental and geochemical parameters effect the microbial community within the tailings samples of site A **(B)**, site B **(C)**, and site C **(D)**. The equation for the average As valence ratio (AVR) is given in Eq. 5.

In the following sections, environmental and geochemical variables ± mineralogical compositions and As valences were related to the overall (Figure 8A) and site-specific (Figures 8B-D) microbial community composition, using non-metric multidimensional scaling (NMDS) plots. The ellipses on Figure 8A correspond to a 95% confidence interval. The *r*^2^-Values for the correlations relating the environmental and geochemical variables to each other are listed in the Supplementary Material Appendix I.

##### Microbial communities and relationships with environmental and geochemical variables

The beta-diversity of the microbial communities between site A and C varied across both the NMDS1 and NMDS2 axes, whereas the composition of site B was dominantly controlled by NMDS1. The microbial community composition at site A compared to that of sites B and C were more similar across the depth profile (indicated by the smaller vs. larger-sized confidence intervals), with the bottom 15 centimeters of site A (*D* = 20, 25, and 30 cm) being very similar (Figure 8A). The composition at site C had a sizeable overlap with site B and small overlap with site A (Figure 8A). Nevertheless, there were three distinct site groupings which are also evident in the plots of the Co# vs. Fe# and S# (Figure 5).

Well-defined relationships were observed when incorporating the environmental and geochemical variables into the bulk microbial community NMDS plot (Figure 8A). The variables controlling the bulk microbial community composition could be divided into two groupings. The first included those with significant *r*^2^ and *p*-Values (>0.5 and <0.05, respectively), including the Fe# and depth. The second included pH, Eh, the concentrations of As, Ca, Co and S_(T)_, and the Co#, of which have slightly less significant *r*^2^ and *p*-Values (<0.5 and between 0.05 and 0.1, respectively) (Supplementary Table A1).

The following observations from Figure 8A can be made:

(1) The Fe# was highest in tailings of site A (specifically in the bottom 15 cm’s) and lowest in tailings from site B and could have a strong influence on the community dissimilarity;
(2) The group 1 (G1; S#, Eh, Fe concentration), group 2 (G2; S_(T)_, concentrations of As and Co), the concentration of Ca, the Co# and the amount of CO_2(T)_ were highest at greater depths (*D* ≥ 15 cm) in the tailings from site C; and
(3) Tailings from site C had the lowest pH values (lowest in *D* = 5 cm).

##### Within-site microbial communities and relationships with environmental and geochemical variables

The relationships between the microbial community composition of sites A to C with environmental and geochemical variables are shown in Figures 8B–D. The composition of the microbial communities at site A was primarily controlled by depth and S_(T)_ all of which have *r*^2^-Values > 0.75 and *p*-Values < 0.05 (Supplementary Table A2). Material at *D* = 30 cm of site A had the highest Eh value which was inversely proportional to the contact pH (*r*^2^ =−0.94; G2 in Figure 8B). The Fe# and S# of group 5 (G5) varied throughout the profile (e.g., both had maxima at *D* = 5 and 15 cm). At *D* = 5 cm, the highest concentration of group 3 (G3, Fe, and S_(T)_) and group 4 (G4, Co# and the concentration of Co) variables occurred (Figure 8B).

In comparison to the microbial communities at site A, the composition of the microbial community at site B were primarily controlled by depth, Eh, pH, and the concentration of Ca (*r*^2^-Values > 0.75 and *p*-Values between 0.005 and 0.08) (Supplementary Table A3). Within tailings from site B, pH, the concentration of As, the average valence of As, and the percent abundances of As^5+^ and As^1–^-species were highly correlated to depth (*r*^2^ > 0.65). The highest contact pH increase occurred within the first 15 cm’s of tailings from site B, with the lowest pH values at *D* = 25 and 30 (Figure 3). The largest decrease in Eh was from *D* = 10 to 15 cm in material from site B. The group 4 (G4) variables (average As valence and the percent abundance of As^5+^-species) were inversely correlated to As^1–^ (*r*^2^ > 0.9), and have the highest values at *D* = 10 and 15 cm (Figure 8C). The concentrations of As and Co were very low at *D* = 30 cm and *D* = 25 and 30 cm, respectively.

The microbial community composition at site C was controlled dominantly by: pH, depth, Eh, concentrations of As and Co, Co#, S#, S_(T)_, and the percent abundances of As^5+^, As^3+^, and As^1–^-species, all of which have *r*^2^ ≥ 0.75 and *p*-Values ≤ 0.06 (Supplementary Table A4). Similar to site B, these variables also significantly correlated with depth (*r*^2^ > 0.8 or *r*^2^ <-−0.8). The Co#, along with the G1 and G2 variables, all increased with depth, with maxima for each variable or group of variables at *D* = 30 cm or *D* = 25 and 30 cm (Figure 8D). The G4 variables with the exception of the average As valence ratio of positive to negative valences (AVR; Eq. 5) had a strong negative correlation with the G1 variables (*r*^2^ ≤-−0.65). The contact pH values were inversely proportional to the CO_2(T)_ concentrations (*r*^2^ =-−0.9), with the highest and lowest values, respectively, at *D* = 5 cm. Maxima of the average As valence, percent abundance of As^5+^-species and the AVR occurred at *D* = 15 cm.


(4) A⁢v⁢e⁢r⁢a⁢g⁢e⁢A⁢s⁢v⁢a⁢l⁢e⁢n⁢c⁢e⁢r⁢a⁢t⁢i⁢o⁢(A⁢V⁢R)=[(A⁢s5++A⁢s3+)(A⁢s1-)]⁢x⁢ 100%

##### Overall trends relating the environmental and geochemical variables to microbial structures

**FIGURE 9:**
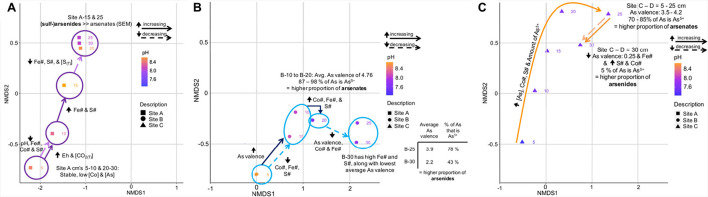
Annotated NMDS plots showing the variation in important environmental and geochemical parameters on the microbial community composition of tailings from site A **(A)**, site B **(B)**, and site C **(C)**. **(B)** has an additional inlet showing the average valence of As and the percent of As that is As^5+^ at *D* = 25 and 30 cm.

The significance of the depth profile on the microbial community composition at site A can be also recognized in the site-specific NDMS plot (Figure 9A). This plot also indicates that other variables such as Eh, CO_2(T)_, pH, Fe#, Co#, S#, and S_(T)_ varied with depth.

The microbial community composition at site B was predominantly controlled by depth and the proportion of As^5+^ with respect to total As; i.e., the proportion of arsenates to arsenides (Figure 9B). The latter value decreased from 87 to 98% at *D* = 10-20 cm to 78% at *D* = 25 and 43% at *D* = 30 cm (Figure 9B). Furthermore, a large increase in the Co# between *D* = 15 and 20 cm and its decrease toward *D* = 30 cm coincided with a change in the microbial community compositions (Figures 5, 9B).

The microbial community composition at site C followed a continuous increase in the concentration of As, Co#, S#, and the amount of As^5+^ (70-85% of As is As^5+^) when transitioning from *D* = 5 cm to *D* = 25 cm (Figure 9C), and correlated strongly with the proportion of arsenates. A sharp decrease in the As valence, the proportion of As^5+^ (5%) and the Fe# between *D* = 25 and 30 cm coincided not only with an increase in the S and Co# but also with a change in the microbial community composition.

## Discussion

We will first discuss environmental parameters controlling microbial community composition before addressing their relationships to the mineralogy and geochemistry of the tailings material at sites A, B, and C of the Cobalt Mining Camp.

### Environmental and/or Geochemical Drivers in Microbial Community Structure

Microbial diversity in mine waste environments can be shaped by various environmental factors and geochemical variables. The characterization of their diversity is important in understanding the biological response to the environmental and geochemical conditions.

The variables associated with governing the microbial structure in the studied tailings were different and more complex than those associated with trends in the mineralogical data (i.e., Co, Fe and S#’s). The following were the most important observations from the relationships between the overall and site-specific microbial communities to the environmental and geochemical variables described,

(1) depth and the Fe# were the primary controllers for the CMC microbial communities;
(2) depth was the only common variable in shaping the structure of the bulk and site-specific microbial communities;
(3) the concentrations of various elements (e.g., As, Co, Fe), pH, Eh and S_(T)_ played a dominant role in determining the microbes present at specific sites;
(4) in site C, the abundances of different As-species (i.e., As^5+^ vs. As^3+^ and As^1–^) strongly correlated to different microbial communities present; and
(5) although the Fe# did not play an important site-specific role in the microbial community structure, it was an important parameter for differentiating the microbial composition between each site.

#### Variation of Microbial Communities With Depth in Each Tailings Profile

The microbial community structure changed, similar to the geochemical and mineralogical composition, with depth at our study sites depicting more similar communities within shallow (0-15 cm) and deeper (16*-*30 cm) samples. Similar trends were observed in As and Sb contaminated soils as well as in slightly acidic mine tailings (e.g., Eilers et al., 2012; Xiao et al., 2017; Ji et al., 2018). Within our relatively shallow depth profiles, an increase in the putative Fe and S-oxidizing genera including *Sulfurifustis*, *Galionella*, and *Thiobacillus* spp. over the 30 cm profile suggested that these shallow profiles still harbored oxic or oxic-anoxic transitions below 15 cm, in accord with the relative high Eh values of 0.25-0.35 V. Facultative chemolithotrophic microaerophiles such as *Gallionella ferruginea* were common in mildly acidic to neutral soil environments (Glasauer et al., 2013) and played an important role in iron oxidation in these systems. A low abundance of putative Fe and S-reducing bacteria increased with depth only at site C, including an abundance of Thermodesulfovibrionia. Sahm et al. (1999); Ravenschlag et al. (2000), Koizumi et al. (2003) identified a similar trend in lake, marine and arctic sediments, where the abundance of these SRBs increased with depth. The difference in abundance of SOB and SRB at sites A and B vs. C may be explained with the development or transition to an anaerobic reduced zone in the bottom layers of site C (i.e., a reduced zone is commonly a zone of a higher abundance of primary arsenides/sulfides relative to an oxidized zone with a higher abundance of arsenates/sulfates).

Similar to the Fe and S-cycling bacteria, those capable of C and N-cycling were also important in tailings, as these were often nutrient limited systems. At sites A and C, the abundance of putative methanotrophs including *Methylobacter*, were also abundant across our 30cm profiles again suggesting the tailings were oxic or at the oxic-anoxic transition at sampled depth. Similarly, van Grinsven et al. (2020) observed a high abundance of facultatively anaerobic and methanotrophic *Methylobacter* sp. in oxygen-limited environments, which they attributed to the coupled methane oxidation to nitrate or nitrite reduction. van Grinsven et al. (2020) also suggested that the greater abundance of *Methylobacter* at greater lake water depth was attributed to nitrate-reducing microorganisms providing *Methylobacter* with a source of nitrite and enabling this genus to thrive in such an environment.

### Roles of Metal and Nutrient-Cycling Bacteria

#### Metal-Cycling Microbial Populations

In a tailings system, lithotrophic organisms commonly occur as the most abundant metabolic groups. These organisms can couple the oxidation of reduced inorganic compounds (such as hydrogen sulfide (HS^–^) and Fe^2+^) to the reduction of a terminal electron acceptor (such as oxygen (O_2_) and sulfate (SO_4_)^2–^) under aerobic and anaerobic conditions. In legacy tailings sites, surrounding influence and inputs from soil and aquatic environmental can introduce micronutrients as well as a mix of metal-cycling and soil nutrient-cycling organisms (e.g., N and C-cyclers).

##### Iron and sulfur-cycling bacteria in neutral-to-alkaline pH conditions

In the neutral-pH tailings of the CMC, the abundance of Fe- and S-bearing minerals strongly affected the diversity of the bulk microbial composition. This relationship was perceived by the site-specific groupings of samples in the plots of Co# vs. Fe# and S#, and the NDMS plots (Figures 5, 8A). Additionally, the occurrence of Fe and S-bearing minerals was correlated with pH, the concentration and average valence of As, and the depth and/or Eh. In acidic pH environments and/or those characterized by AMD, Fe- and S-cycling bacteria were well-characterized (e.g., Kock and Schippers, 2008; Guan et al., 2017). However, in metal(loid)-rich, Fe- and S-poor tailings environments with a neutral-to-alkaline pH, the role of these microbes were not as well understood.

In general, the CMC tailings had a higher abundance of FeOB and SOB relative to IRB and SRB through the 30 cm profile. Many of the putatively identified FeOB and SOB (e.g., *Sideroxydans*, *Sulfuricella*, and *Thiobacillus*) are known to grow under neutral pH, and are dependent on nitrate as either a chemical oxidant (under anaerobic conditions) or as an electron acceptor (Haaijer et al., 2012; Bryce et al., 2018). The abundance of *Sulfurifustis* sp. initially isolated from lake water (Umezawa et al., 2016), suggested several organisms are driving the oxidation of reduced sulfur compounds under neutral conditions. The diversity and activity of IRB are not well-known in neutral/alkaline S-rich mine tailings environments as they are potentially competing with SRB for common electron donors (Praharaj and Fortin, 2008). Interestingly, both SOB (*Thiobacillus* and *Sulfurifustis* spp.) and putative SRB (class Thermodesulfovibrionia) co-occurred in tailings at site C at 30cm suggesting the transition between sulfur oxidation and reduction were present at this depth. This coincided with the highest total S, As and Co concentrations in these materials suggesting these genera were likely involved in the biotic transformation of arsenides (discussed below).

##### Carbon-cycling bacteria

In the CMC tailings, the total C content affected the microbial community composition at site B, and likely linked to the revegetation activities around the tailings in the late 1990’s (Percival et al., 2004; Dumaresq, 2009). Here, the CO_2(T)_ content decreased from 5 wt% at the top to 3 wt% at the bottom (Figure 3D) and resulted most likely in a much higher diversity of common soil microorganisms in the top sample (B-05), including the abundance of heterotrophic groups including Rhizobiales, Rhodobacterales, and Sphingomonadales commonly found in soils (Garris et al., 2018). These bacterial orders are also commonly found in contaminated soils and can help promote bioremediation by establishing nutrient cycling for C and N (Garris et al., 2018).

Additionally, methanotrophic bacteria formed a significant component of the microbial community within the tailings suggesting some influence of organic/methanogenic environments underlying the tailings deposits. Methanotrophic bacteria, such as those of the genera *Methylocystis*, *Methylocapsa*, and *Methylobacter* were identified in metal and/or As-contaminated environments (e.g., those impacted by AMD, Gorra et al., 2012; Park et al., 2018). Due to the metal (Cu) dependence and high metal binding affinity of the methane monooxygenase (MMO) enzyme found in all methanotrophs, these bacteria potentially played a role in the mobility of a variety of metals (Jenkins et al., 1994; Choi et al., 2006; Pandey et al., 2014) and appeared to be well adapted to the conditions found at all three CMC sites.

##### Nitrogen-cycling bacteria

There was a high relative abundance of putative N-cycling bacteria at sites B and C (e.g., *Gaiella* and *Nitrosospira* spp.) and those that couple nitrate-reduction to Fe and/or S-oxidation (e.g., *Thiobacillus* spp.). The high abundance of these microbes could be due to the implementation of cyanide during ore-processing (Anderson, 1993; Dumaresq, 1993), as N and As can co-occur in these environments due to biotic (over abiotic) oxidation of arseno-sulfide minerals such as arsenopyrite (Bissen and Frimmel, 2003; EPA, 2003; Dave et al., 2008). It is however more likely that N-cycling bacteria colonized the tailings due to natural environmental inputs of N, for example from N-fixation processes occurring in the surface tailings layers influenced by proximal soil/wetland or lake environments. Interestingly, putative nitrogen fixers including *Methylocapsa* sp. (Dedysh et al., 2002) and *Methylovirgula* sp. were lower in abundance at the site A tailings profile, suggesting again that oxic conditions and nitrogen fixation occurred to *D* = 30cm and were linked to CH_4_ oxidation. Ammonium-oxidation by nitrifying bacteria (e.g., *Nitrospira*) could also be cycled in the tailings and occurred in sediments with observed As^3+^-oxidation under oxic conditions at neutral pH, but in in the absence of known As-oxidizers (Papirio et al., 2014).

#### Microbial Community Composition vs. the Mineralogy and Average Valence of Arsenic

The biogeochemical cycling of As is important in terms of ecological toxicity and bioremediation processes. Microbes have a variety of roles in As-cycling, such as the biomethylation of As (e.g., arsenite to trimethylarsine), the reduction of arsenates or arsenites, and the oxidation of arsenides or arsenites (Gadd, 2010).

The most apparent and distinct change in Arsenic mineralogy and microbial structure occurred at site C where the mineralogical transition from the reduced (arsenides) to oxidized (arsenates) zone correlated with a decrease in the abundance of S and As-reducing bacteria.

At site B, the higher abundance of arsenates and arsenides at shallower and greater depth, correlated well with a higher abundance of methanotrophic bacteria and SOB and FeOB, respectively. Similarly, the higher abundance of (sulf)arsenides at site A correlated well with increasing abundances of methanotrophic bacteria, SOB and FeOB at greater depths.

These observations indicated that tailings material enriched or depleted in (sulf)arsenides or arsenates had distinct microbial community composition but they also suggested that the latter compositions were not solely controlled by the type of As-bearing minerals.

A significant correlation between the microbial community composition and the average valence of As was only identified at site C. At both sites B and C, a lower average As valence occurred at greater depths, with the key difference being that Fe and S-oxidizing bacteria at site B were in larger abundance, whereas at site C, S, and (potential) As-reducing bacteria were in higher abundance. However, it is possible that some of the SOB was able to reduce As. For example, two of the identified SOB (*Sulfurifustis*, and *Thiobacillus* spp.), were able to couple S-oxidation and As-reduction, and *Thiobacillus* spp. using nitrate as the preferred electron donor (Hollibaugh et al., 2006; Guan et al., 2017). Additionally, *Nocardioides* spp. were capable of As^5+^-reduction in neutral conditions (Bagade et al., 2016). The putative SRB belonging to the class Thermodesulfovibrionia, that occurred in abundance in the bottom layer of site C, and were capable of simultaneously reducing sulfate and arsenate species, resulting in the precipitation of biogenic Fe-As-S minerals (Zacarías-Estrada et al., 2020). Kelly et al. (2007) proposed a similar process for the sequestration of metal(loid)s in wetland sediments of the CMC without providing any mineralogical evidence.

#### Iron-Mobilization in Neutral-pH Tailings Conditions

Iron-(hydr)oxides are the most thermodynamically stable Fe-phase under near-neutral to alkaline conditions, with their formation being inhibited by the presence of IRB (Revesz et al., 2015). Courchesne et al. (2021) showed that Co-Ni-Zn arsenates rather than Fe-(hydr)oxides could replace scorodite under circumneutral conditions. The authors argued that the replacement of scorodite was promoted by the formation of Fe-carbonate complexes and the sequestration of Fe through its intercalation in the interlayers of 2:1 clay minerals.

The analyses of the microbial community compositions at sites A, B and C indicated that the dissolution of scorodite and the mobilization of Fe as Fe^2+^-species may have also been promoted by putative IRB such as those of the genera *Bacillus* and *Paenibacillus* and *Rhodoferax* genera (Yi et al., 2012; Li et al., 2020). These genera are however phenotypically and genotypically diverse and widespread in the environment (Porwal et al., 2009; Grady et al., 2016). Nevertheless, certain species within these genera, along with many aerobic bacteria, are capable of indirect Fe-reduction through siderophore production in Fe-limited conditions, or simply by allowing Fe transport through the cell wall (Albrecht-Gary and Crumbliss, 1998; Raza and Shen, 2010). Additionally, an anaerobic Fe^3+^-reducing bacterium, *Rhodoferax ferrireducens* (Finneran et al., 2003), appeared in the more reduced conditions within the bottom layer of site C, but in very low abundance (∼1.6%) providing evidence for potential IRB-mediated mobilization of Fe within the bottom tailings material of the CMC whereas the majority of the site A and B profiles were dominated by oxidizing species. The microbially mediated reduction of Fe^3+^ were likely limited to larger micrometer-size pore spaces within the tailings material, whereas the observed mineral replacement reactions of scorodite by Co, Ni, Zn-arsenates and the potential complexation of Fe^3+^ by carbonate species could occur in micro- and nanopores (Courchesne et al., 2021).

### Summary of Microbes Potentially Involved in As-Cycling for Use in Bioleaching of As-Rich Neutral-pH Tailings

Overall, the microbial structure of the CMC tailings consisted of some Fe and S-cycling bacteria, and nitrate reducing and methanotrophic bacteria, some of which may be indirectly involved in the biogeochemical cycling of As (i.e., the use of (AsO_4_)^2–^ as a terminal electron acceptor) or those found in As (or other metal) contaminated sites. Furthermore, the occurrence of novel neutrophilic, As-cycling bacteria within the characterized CMC tailings were likely present, due to the ∼ 25–55% uncharacterized genera and >85% uncharacterized species in the amplicon data. Many of the identified microbial genera in the CMC tailings are known to have As resistant genes, encoded by the *ars* gene, such as genera *Bacillus*, *Sphingomonas* and *Sulfuricella*, as well as additional previously unmentioned genera such as *Xylophilus* (Jackson et al., 2005). Moreover, the findings by Jackson et al. (2005) supported the widespread occurrence of bacteria resistant to As in the environment, even within nearly pristine soils. Guan et al. (2017) also concluded that microbes are capable of building up a tolerance to As over time. It is therefore likely, that enrichments and isolation of bacteria from these materials will help identify those with high As tolerance and potential of mineral transformation (such as liberation of Co through bioleaching).

## Conclusion

This multi-variate and multi-disciplinary study on the geochemical, mineralogical, and microbiological characteristics of the tailings at the CMC provided an in-depth understanding of the tailings materials that will assist in the development of neutrophilic microbial consortia capable of mobilizing the critical element cobalt and the toxic element arsenic from arsenides and arsenates through applications like bioleaching. This study added valuable biogeochemical knowledge to tailings systems characterized by neutral-to-alkaline mine drainage. The main findings of which include:

(1) Sites A and B in this study shared a similar geochemistry and mineralogy to that observed by Courchesne et al. (2021);
(2) Site C was geochemically and mineralogically dissimilar to sites A and B, as site C had distinct reduced and oxidized zones, as observed in more acidic mine tailings environments;
(3) The groupings observed of the Co# vs. Fe# and S# between each site, correlated well with site-specific microbial groupings;
(4) The abundance of microbial communities involved in cycling Fe, S, N and C correlated with depth, i.e., the occurrence of SRB at greater depths coincided with a low average valence of As;
(5) The presence of FeOB and SOB at greater depths within the CMC tailings could be due to the presence of (sulf-)arsenides;
(6) Samples at site B with low Co#’s and high Fe and/or S#’s had a proportionally higher abundance of FeOB or SOB, due to the abundance of (sulf-)arsenides, whereas at site C, a low Fe# and high Co and S#’s showed an abundance of (sulf)-arsenides, sulfides, and SRB;
(7) At individual sites, high Co#’s did not correlate with a higher abundance of potential As-cycling bacteria; and
(8) The low abundance or absence of IRB and SRB at the bottom of site A and B suggested that the tailings remained largely oxic or at the oxic-anoxic transition up to 30cm.

## Data Availability Statement

The datasets presented in this study can be found in online repositories. The names of the repository/repositories and accession number(s) can be found below: NCBI BioProject, accession no: PRJNA700089.

## Author Contributions

BC, MS, and NM were involved in the design of the study. MS and NM supervised the study. MS conducted a portion of the scanning electron microscopy and X-ray diffraction work. BC wrote the first draft and finalized the manuscript. All authors contributed to the writing of the manuscript and approved submission.

## Conflict of Interest

The authors declare that the research was conducted in the absence of any commercial or financial relationships that could be construed as a potential conflict of interest.

## Publisher’s Note

All claims expressed in this article are solely those of the authors and do not necessarily represent those of their affiliated organizations, or those of the publisher, the editors and the reviewers. Any product that may be evaluated in this article, or claim that may be made by its manufacturer, is not guaranteed or endorsed by the publisher.

## Footnotes

1
https://jgi.doe.gov/data-and-tools/bbtools/bb-tools-user-guide/bbduk-guide/
