# Supplementary material for: Relationships Between the Microbial Composition and the Geochemistry and Mineralogy of the Cobalt-Bearing Legacy Mine Tailings in Northeastern Ontario
Source: Front Microbiol. 2021 Sep 17;12:660190. doi: 10.3389/fmicb.2021.660190 (PMC8485068; doi:10.3389/fmicb.2021.660190)
Supplement: Supplementary file 1 [file Data_Sheet_1.docx]

# Supplementary Material Appendix A: Mineralogy of the Cobalt mining camp

| Supplementary Table A1. Mineralogy identified from the ore and tailings of the Cobalt mining camp. | | | |
| --- | --- | --- | --- |
| **Ore Mineralogy** | | | |
| **Mineral name** | **Mineral formula** | **Mineral name** | **Mineral formula** |
| Native silver | Ag^0^ | Acanthite | AgS_2_ |
| Native bismuth | Bi^0^ | Proustite | Ag_3_AsS_3_ |
| Dyscrasite | Ag_3_Sb | Stephanite | Ag_5_SbS_4_ |
| Argentite | Ag_2_S | Allargentum | Ag_1-x_S_x_ (x=0.09-0.16) |
| Cobaltite | CoAsS (**) | Chapmanite | Fe_2_Sb(Si_2_O_5_)O_3_(OH) |
| Skutterudite | (Co,Fe,Ni)As_2-3_ (**) | Langisite | (Co,Ni)As |
| Niccolite | NiAs (**) | Larosite | (Cu,Ag)_21_(Pb,Bi)_2_S_13_ |
| Mckinstryite | Ag_5-x_Cu_3+x_S_4_ (x=0-0.28) | Gersdorffite | NiAsS (**) |
| Pararammelsbergite | NiAs_2_ | Bismuthinite | Bi_2_S_3_ (**) |
| Arsenopyrite | FeAsS (**) | Chalcopyrite | CuFeS_2_ (**) |
| Cobaltoan arsenopyrite | (Fe,Co)AsS (**) | Argentobismutite | AgBiS_2_(**) |
| Argentopyrite | AgFe_2_S_3_ (**) | Safflorite | (Co,Ni,Fe)As_2_ (**) |
| Löllingite | FeAs_2_ (**) |  |  |
| **Tailings Mineralogy** | | | |
| **Mineral name** | **Mineral formula** | **Mineral name** | **Mineral formula** |
| Albite | NaAlSi_3_O_8_ | Scorodite | Fe^3+^(AsO_4_)⋅2H_2_O |
| Quartz | SiO_2_ | Arsenohopeite | Zn_3_(AsO_4_)_2_⋅2H_2_O |
| Anorthite | CaAl_2_Si_2_O_8_ | Goethite | α-Fe^3+^O(OH) |
| Dolomite | CaMg(CO_3_)_2_ | Calcite | CaCo_3_ |
| Clinochlore | Mg_5_Al(AlSi_3_O_10_)(OH)_8_ | Alunite | KAl_3_(SO_4_)_2_(OH)_6_ |
| Erythrite | Co_3_(AsO_4_)_2_⋅8H_2_O | Annabergite | Ni_3_(AsO_4_)_2_⋅8H_2_O |
| Cobaltoan olivenite | (Cu,Co)_2_AsO_4_(OH) | Titanite | CaTi(SiO_4_)O |
| Stilbite | NaCa_4_[Al_9_Si_27_O_72_⋅nH_2_O | | |
| Roselite | Ca_2_(Co^2+^,Mg)(AsO_4_)_2_⋅2H_2_O | | |
| Ferropargasite | NaCa_2_Fe_4_Al(Si_6_Al_2_)O_22_(OH)_2_ | | |
| Muscovite | (K,Ca,Na)(Al,Mg,Fe)_2_(Si,Al)_4_O_10_(OH)_2_ | | |
| Illite | (K,H_3_O)Al_2_Si_3_AlO_10_(OH)_2_ | | |
| Magnesioriebeckite | (Na,Ca)_2_(Mg,Fe)_5_Si_8_O_22_(OH)_2_ | | |
| Magnesioarfvedsonite | (Na)(Na_2_)(Mg_4_Fe^3+^)(Si_8_O_22_)(OH)_2_ | | |
| Epidote | Ca_2_(Al_2_Fe^3+^)(Si_2_O_7_)(SiO_4_)O(OH) | | |

** occurs in ore and tailings. All mineral formulae were retrieved from https://www.mindat.org/.

# Supplementary Material Appendix B: X-ray Diffractograms

|  |
| --- |
| Supplementary Figure B1. X-ray diffraction patterns from site A D = 15 (a), D = 20 (b) and D = 25 (c). |

|  |
| --- |
| Supplementary Figure B2. X-ray diffraction patterns from site B D = 10 (a), D = 25 (b) and D = 30 (c). |

|  |
| --- |
| Supplementary Figure B3. X-ray diffraction patterns from site C D = 20 (a), D = 25 (b) and D = 30 (c). |

# Supplementary Material Appendix C: Raw Sequence Data and Rarefaction Curve

| 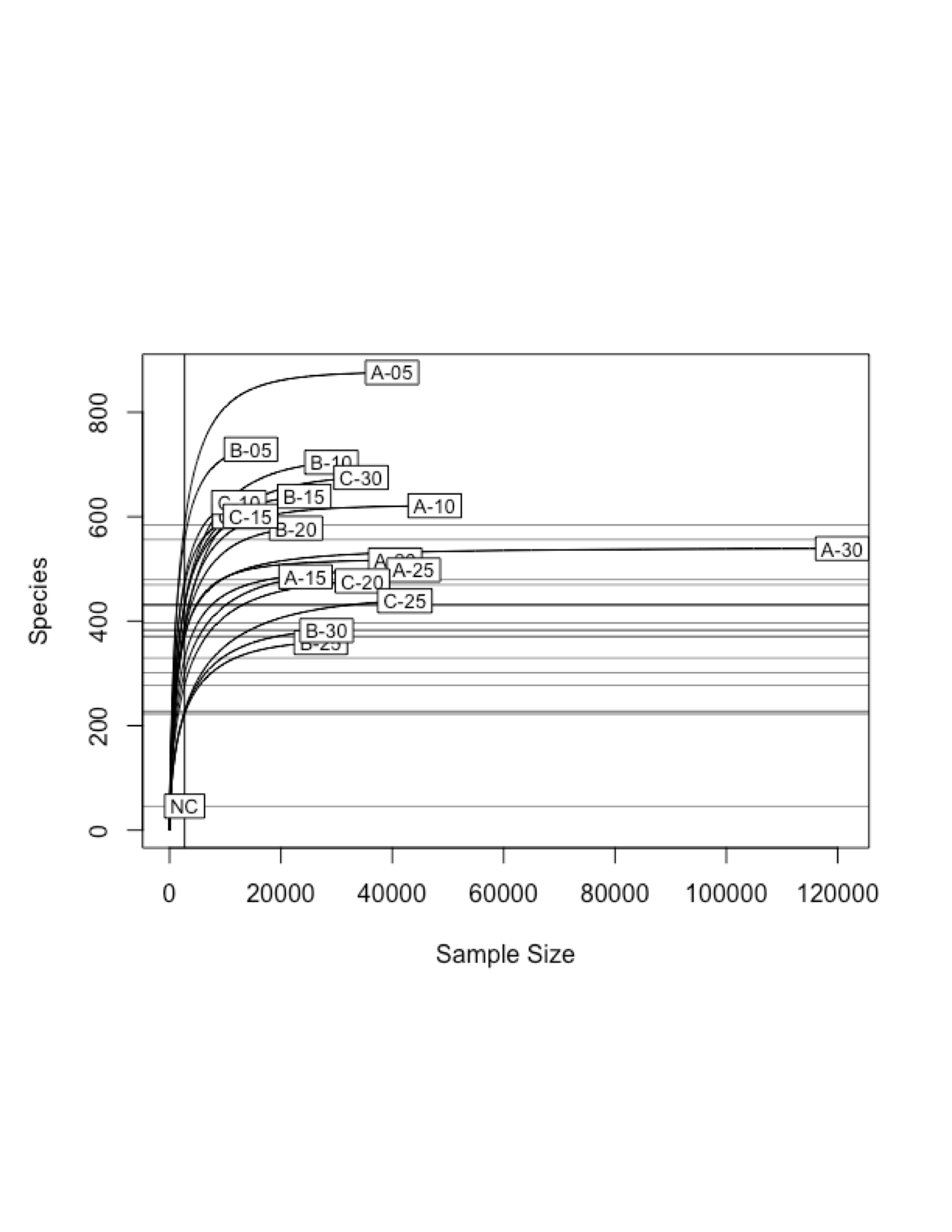 |
| --- |
| Supplementary Figure C1. A rarefaction curve plot of tailings samples analyzed for 16S rRNA gene sequencing. The sample size (or sequencing depth) is on the X-axis, with species count on the Y-axis. Samples that have a curve that has plateaued means that the sequences were well-recovered, with some having a low species count meaning that there could have been a low DNA yield or the samples are not well diverse. Samples that have a curve that is beginning to plateau means that more sample diversity could have been captured, with (possibly) better extraction methods. |

| Supplementary Table C1. A chart of the raw bacterial sequence data showing the total number of sequences, the percent of sequences filtered out, and the percent of sequences retained. | | | |
| --- | --- | --- | --- |
| **Sample ID** | **Total Number of Sequences** | **% Filtered** | **% Retained** |
| A-05 | 49153 | 4.5 | 95.5 |
| A-10 | 54213 | 3.9 | 96.1 |
| A-15 | 27301 | 2.5 | 97.5 |
| A-20 | 44540 | 3.3 | 96.7 |
| A-25 | 48401 | 3.2 | 96.8 |
| A-30 | 134261 | 3.9 | 96.1 |
| B-05 | 19960 | 3.1 | 96.9 |
| B-10 | 36860 | 3.5 | 96.5 |
| B-15 | 31114 | 3.2 | 96.8 |
| B-20 | 28089 | 3.5 | 96.5 |
| B-25 | 33786 | 3.5 | 96.5 |
| B-30 | 34573 | 3.3 | 96.7 |
| C-05 | 17099 | 3.5 | 96.5 |
| C-10 | 17153 | 3.0 | 97.0 |
| C-15 | 21281 | 2.8 | 97.2 |
| C-20 | 53910 | 3.2 | 96.8 |
| C-25 | 52945 | 2.8 | 97.2 |
| C-30 | 39746 | 2.6 | 97.4 |
| NC | 3202 | 3.4 | 96.6 |

# Supplementary Material Appendix D: XANES Spectra

|  |
| --- |
| Supplementary Figure D1. The triplicate averaged XANES spectra for the standards used. |

| 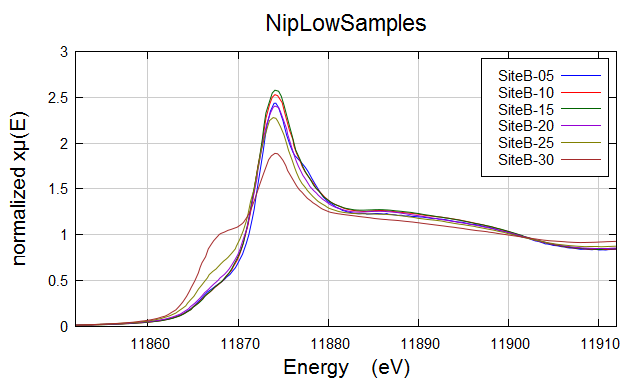 |
| --- |
| Supplementary Figure D2. The triplicate averaged XANES spectra for site B tailings samples. |

| 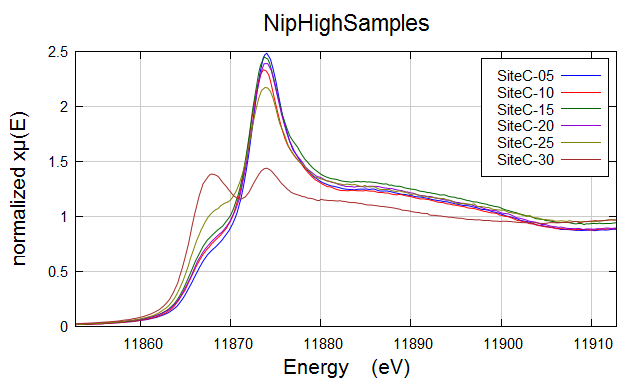 |
| --- |
| Supplementary Figure D3. The triplicate averaged XANES spectra for site C tailings samples. |

# Supplementary Material Appendix E: Analysis of Similarities (ANOSIM)

| 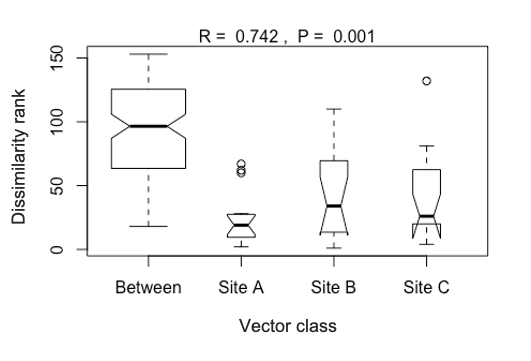 |
| --- |
| Supplementary Figure E1. A graph resulting from the analysis of similarities (ANOSIM) test. The analysis was performed to identify dissimilarities between and within sites, using the microbial and geochemical data. An R-value of 0.742 with a p-value of 0.001 indicates a strong, statistically significant difference in the microbial communities based on the site grouping |

# Supplementary Material Appendix F: Bulk Chemical Data

| Supplementary Table F1. Table showing the values or concentrations of the environmental and geochemical variables in each tailings sample. | | | | | | | | | | | | | |
| --- | --- | --- | --- | --- | --- | --- | --- | --- | --- | --- | --- | --- | --- |
| **ID** | **pH** | **Eh** (V) | **Ag** (ppm) | **As** (wt%) | **Ca** (wt%) | **Co** (wt%) | **Fe** (wt%) | **CO_2(T)_** (wt%) | **S_(T)_** (wt%) | **Co#** | **Fe#** | **S#** |  |
| A-05 | 8.48 | 0.28 | 10.61 | 0.07 | 4.31 | 0.03 | 7.40 | 1.44 | 0.09 | 6.83 | 81.72 | 5.10 |  |
| A-10 | 8.32 | 0.29 | 44.03 | 0.05 | 3.95 | 0.02 | 7.14 | 1.84 | 0.08 | 4.10 | 78.36 | 3.85 |  |
| A-15 | 8.7 | 0.27 | 27.75 | 0.03 | 4.04 | 0.01 | 6.80 | 1.75 | 0.08 | 3.89 | 81.31 | 4.69 |  |
| A-20 | 8.57 | 0.27 | 43.43 | 0.07 | 4.28 | 0.02 | 7.10 | 1.65 | 0.08 | 5.57 | 80.73 | 4.40 |  |
| A-25 | 8.06 | 0.29 | 52.31 | 0.04 | 4.17 | 0.02 | 5.10 | 1.63 | 0.08 | 4.15 | 74.92 | 4.26 |  |
| A-30 | 8.05 | 0.30 | 47.85 | 0.05 | 3.85 | 0.02 | 7.43 | 1.82 | 0.07 | 4.17 | 79.96 | 3.82 |  |
| B-05 | 8.63 | 0.25 | 78.28 | 0.42 | 3.19 | 0.11 | 5.58 | 4.861 | 0.07 | 17.1 | 66.46 | 2.53 |  |
| B-10 | 8.08 | 0.27 | 75.83 | 0.39 | 2.76 | 0.12 | 6.26 | 3.102 | 0.07 | 15.7 | 67.44 | 2.17 |  |
| B-15 | 8.14 | 0.27 | 78.81 | 0.36 | 2.70 | 0.13 | 7.95 | 2.826 | 0.07 | 11.6 | 66.00 | 1.68 |  |
| B-20 | 7.7 | 0.26 | 109.5 | 0.38 | 2.47 | 0.14 | 5.85 | 4.848 | 0.17 | 16.8 | 67.89 | 5.82 |  |
| B-25 | 7.84 | 0.26 | 96.56 | 0.40 | 2.47 | 0.11 | 6.13 | 2.335 | 0.06 | 15.6 | 67.02 | 2.01 |  |
| B-30 | 7.77 | 0.26 | 108.5 | 0.29 | 2.81 | 0.10 | 6.71 | 3.259 | 0.14 | 11.1 | 65.24 | 3.69 |  |
| C-05 | 7.23 | 0.36 | 15.17 | 0.88 | 4.12 | 0.37 | 10.51 | 6.498 | 0.19 | 19.9 | 67.07 | 3.50 |  |
| C-10 | 7.58 | 0.36 | 26.24 | 1.15 | 5.22 | 0.46 | 12.40 | 4.848 | 0.17 | 22.6 | 68.70 | 2.94 |  |
| C-15 | 7.53 | 0.36 | 8.17 | 1.29 | 3.97 | 0.46 | 12.74 | 4.969 | 0.22 | 24.5 | 69.79 | 3.85 |  |
| C-20 | 7.66 | 0.36 | 9.72 | 1.37 | 4.05 | 0.51 | 11.80 | 4.995 | 0.26 | 27.3 | 69.78 | 4.81 |  |
| C-25 | 7.67 | 0.36 | 27.06 | 1.93 | 4.33 | 0.63 | 11.97 | 5.154 | 0.29 | 32.8 | 69.09 | 5.05 |  |
| C-30 | 7.6 | 0.34 | 41.30 | 1.94 | 6.96 | 1.20 | 11.43 | 4.676 | 0.56 | 37.9 | 68.34 | 9.56 |  |

| Supplementary Table F2. Table showing the average As valence, the percent abundances of As-species and the ratio of positive to negative As species (AVR) (Equation 5). | | | | | |
| --- | --- | --- | --- | --- | --- |
| **Sample ID** | **Average As valence** | **As^5+^ (%)** | **As^3+^ (%)** | **As^1-^ (%)** | **AVR** |
| B-05 | 4.27 | 87.77 | 0.00 | 12.23 | 7.17 |
| B-10 | 4.90 | 94.80 | 5.2 | 0.00 | 0.00 |
| B-15 | 4.92 | 96.10 | 3.90 | 0.00 | 0.00 |
| B-20 | 4.47 | 86.03 | 7.63 | 6.33 | 14.79 |
| B-25 | 3.93 | 71.10 | 17.10 | 11.83 | 7.45 |
| B-30 | 2.18 | 43.97 | 13.57 | 42.47 | 1.35 |
| C-05 | 4.04 | 73.27 | 16.03 | 10.70 | 8.34 |
| C-10 | 3.71 | 62.80 | 23.6 | 13.63 | 6.34 |
| C-15 | 4.28 | 68.97 | 28.53 | 2.50 | 39.00 |
| C-20 | 3.86 | 67.20 | 20.77 | 12.03 | 7.31 |
| C-25 | 3.52 | 51.70 | 31.07 | 17.20 | 4.81 |
| C-30 | 0.25 | 11.83 | 13.57 | 74.60 | 0.34 |

# Supplementary Material Appendix G: Additional SEM results from sites B and C

| 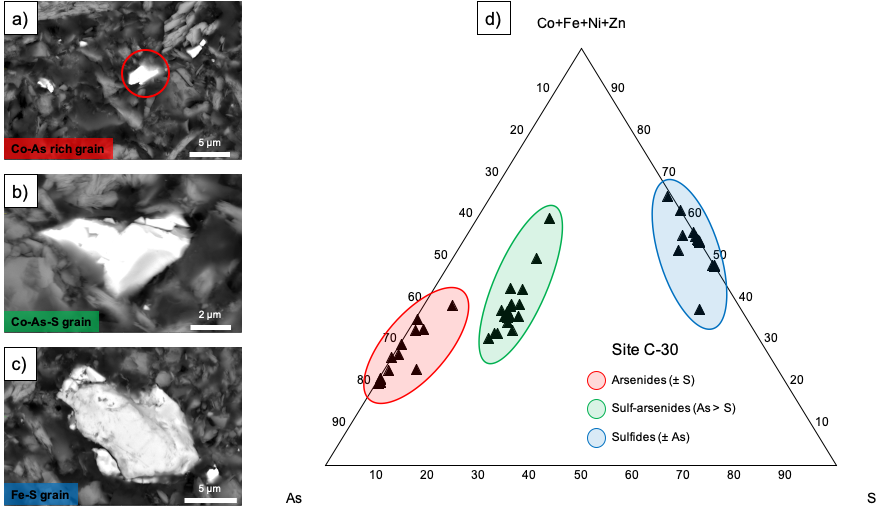 |
| --- |
| Supplementary Figure G1. a) – c) SEM photomicrographs from D = 30 cm at site C, a) Co-arsenide, b) Co-sulfarsenide and c) Fe-sulfide, and d) a ternary diagram of the three different As-species identified on the SEM. |

# Supplementary Material Appendix H: Additional Taxonomic Plots

| 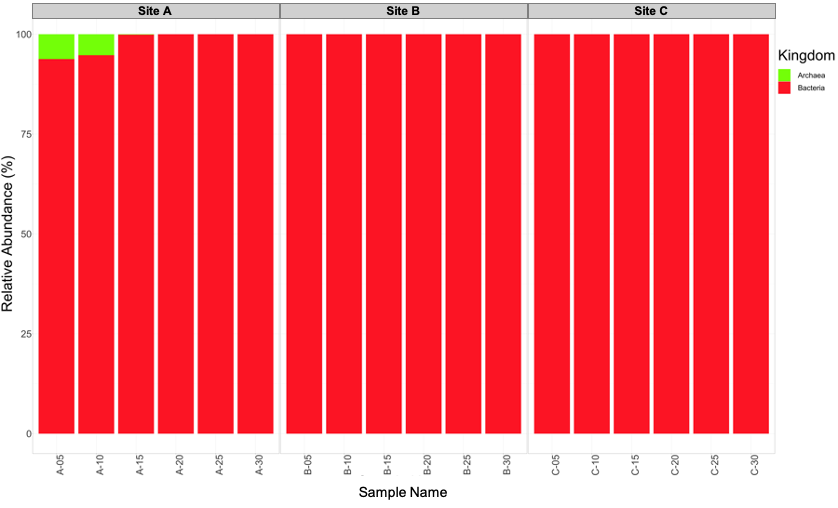 |
| --- |
| Supplementary Figure H1. Normalized relative abundance taxonomic bar plot of microbial communities at the Kingdom level. |

| 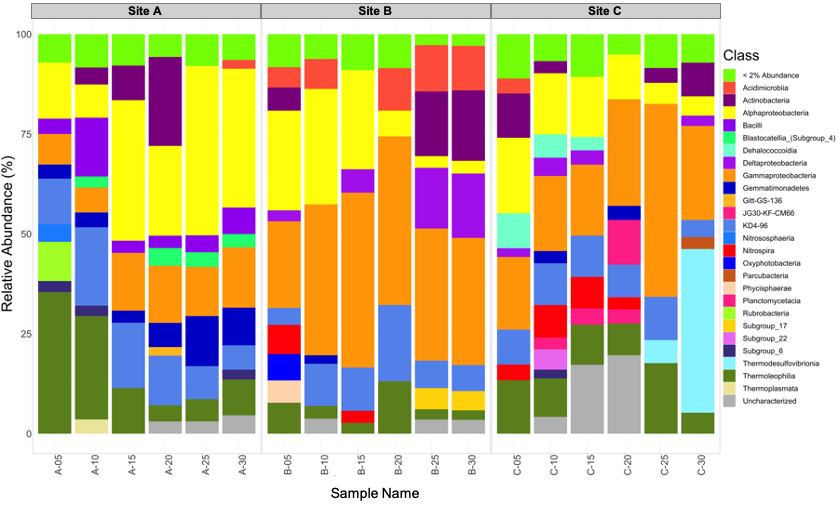 |
| --- |
| Supplementary Figure H2. Normalized relative abundance taxonomic bar plot of microbial communities at the Class level. |

| 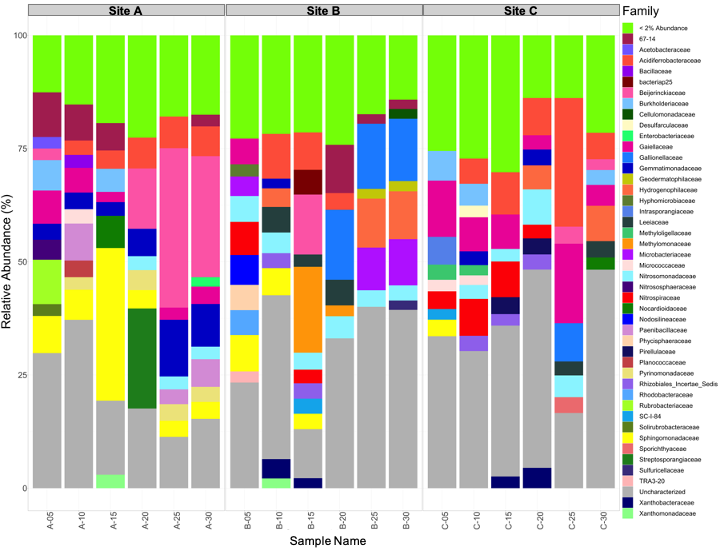 |
| --- |
| Supplementary Figure H3. Normalized relative abundance taxonomic bar plot of microbial communities at the Family level. |

| 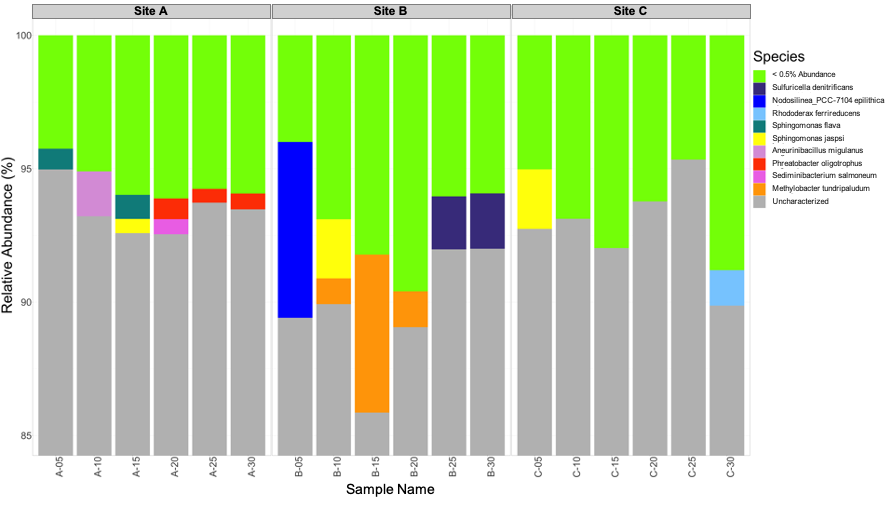 |
| --- |
| Supplementary Figure H4. Normalized relative abundance taxonomic bar plot of microbial communities at the Species level. |

# Supplementary Material Appendix I: Correlation Matrices

| Supplementary Table I1. Correlation matrix showing the correlations of environmental and geochemical variables to each other for  material from site A. | | | | | | | | | | | | | |
| --- | --- | --- | --- | --- | --- | --- | --- | --- | --- | --- | --- | --- | --- |
| **SITE A** | pH | Depth | Eh (V) | Ag (ppm) | As (wt%) | Ca (wt%) | Co (wt%) | Fe (wt%) | CO_2(T)_ (wt%) | S_(T)_ (wt%) | Co# | Fe# | S# |
| pH | 1 | -0.61 | -0.94 | -0.63 | 0.26 | 0.43 | -0.025 | 0.37 | -0.24 | 0.39 | 0.33 | 0.71 | 0.64 |
| Depth | -0.61 | 1 | 0.34 | 0.78 | -0.44 | -0.41 | -0.57 | -0.350 | 0.42 | -0.84 | -0.52 | -0.41 | -0.59 |
| Eh (V) | -0.94 | 0.34 | 1 | 0.45 | -0.21 | -0.49 | 0.16 | -0.22 | 0.25 | -0.2 | -0.28 | -0.63 | -0.61 |
| Ag (ppm) | -0.63 | 0.78 | 0.45 | 1 | -0.5 | -0.46 | -0.6 | -0.45 | 0.63 | -0.85 | -0.68 | -0.71 | -0.86 |
| As (wt%) | 0.26 | -0.44 | -0.21 | -0.5 | 1 | 0.6 | 0.74 | 0.57 | -0.62 | 0.73 | 0.94 | 0.53 | 0.42 |
| Ca (wt%) | 0.43 | -0.41 | -0.49 | -0.46 | 0.6 | 1 | 0.61 | -0.18 | -0.9 | 0.64 | 0.77 | 0.13 | 0.77 |
| Co (wt%) | -0.025 | -0.57 | 0.16 | -0.6 | 0.74 | 0.61 | 1 | 0.15 | -0.81 | 0.9 | 0.85 | 0.13 | 0.54 |
| Fe (wt%) | 0.37 | -0.35 | -0.22 | -0.45 | 0.57 | -0.18 | 0.15 | 1 | 0.11 | 0.33 | 0.38 | 0.85 | 0.055 |
| CO_2(T)_ (wt%) | -0.24 | 0.42 | 0.25 | 0.63 | -0.62 | -0.9 | -0.81 | 0.11 | 1 | -0.78 | -0.85 | -0.18 | -0.84 |
| S_(T)_ (wt%) | 0.39 | -0.84 | -0.2 | -0.85 | 0.73 | 0.64 | 0.9 | 0.33 | -0.78 | 1 | 0.86 | 0.43 | 0.75 |
| Co# | 0.33 | -0.52 | -0.28 | -0.68 | 0.94 | 0.77 | 0.85 | 0.38 | -0.85 | 0.86 | 1 | 0.49 | 0.69 |
| Fe# | 0.71 | -0.41 | -0.63 | -0.71 | 0.53 | 0.13 | 0.13 | 0.85 | -0.18 | 0.43 | 0.49 | 1 | 0.49 |
| S# | 0.64 | -0.59 | -0.61 | -0.86 | 0.42 | 0.77 | 0.54 | 0.055 | -0.84 | 0.75 | 0.69 | 0.49 | 1 |

| Supplementary Table I2. A correlation matrix showing the correlations of environmental and geochemical variables to each other for material from site B. | | | | | | | | | | | | | | | | | | | |
| --- | --- | --- | --- | --- | --- | --- | --- | --- | --- | --- | --- | --- | --- | --- | --- | --- | --- | --- | --- |
| **SITE B** | pH | Depth | Eh (V) | Ag (ppm) | As (wt%) | Ca (wt%) | Co (wt%) | Fe (wt%) | CO_2(T)_ (wt%) | S_(T)_ (wt%) | Co# | Fe# | S# | As Val | As^5+^ | As^3+^ | As^1-^ | AVR |  |
| pH | 1 | -0.85 | -0.39 | -0.8 | 0.55 | 0.85 | -0.21 | -0.12 | 0.31 | -0.6 | 0.26 | -0.15 | -0.52 | 0.37 | 0.48 | -0.16 | -0.29 | -0.22 |  |
| Depth | -0.85 | 1 | 0.058 | 0.84 | -0.72 | -0.6 | -0.23 | 0.2 | -0.42 | 0.47 | -0.5 | -0.3 | 0.33 | -0.72 | -0.81 | 0.019 | 0.65 | 0.074 |  |
| Eh (V) | -0.39 | 0.058 | 1 | -0.14 | -0.31 | -0.49 | 0.56 | 0.66 | -0.4 | 0.043 | -0.41 | 0.15 | -0.046 | 0.4 | 0.35 | 0.41 | -0.42 | -0.41 |  |
| Ag (ppm) | -0.8 | 0.84 | -0.14 | 1 | -0.54 | -0.52 | 0.007 | -0.19 | 0.13 | 0.83 | -0.12 | -0.02 | 0.77 | -0.65 | -0.71 | -0.28 | 0.61 | 0.53 |  |
| As (wt%) | 0.55 | -0.72 | -0.31 | -0.54 | 1 | 0.15 | 0.26 | -0.54 | 0.26 | -0.49 | 0.86 | 0.67 | -0.24 | 0.71 | 0.7 | 0.35 | -0.71 | 0.37 |  |
| Ca (wt%) | 0.85 | -0.6 | -0.49 | -0.52 | 0.15 | 1 | -0.5 | -0.17 | 0.4 | -0.3 | 0.044 | -0.44 | -0.3 | -0.098 | 0.051 | -0.61 | 0.19 | -0.32 |  |
| Co (wt%) | -0.21 | -0.23 | 0.56 | 0.007 | 0.26 | -0.5 | 1 | 0.1 | 0.3 | 0.31 | 0.3 | 0.67 | 0.41 | 0.71 | 0.68 | 0.37 | -0.72 | 0.44 |  |
| Fe (wt%) | -0.12 | 0.2 | 0.66 | -0.19 | -0.54 | -0.17 | 0.1 | 1 | -0.58 | -0.19 | -0.85 | -0.53 | -0.4 | 0.065 | 0.054 | 0.36 | -0.069 | -0.65 |  |
| CO_2(T)_ (wt%) | 0.31 | -0.42 | -0.4 | 0.13 | 0.26 | 0.4 | 0.3 | -0.58 | 1 | 0.53 | 0.54 | 0.28 | 0.65 | 0.093 | 0.2 | -0.64 | -0.019 | 0.63 |  |
| S_(T)_ (wt%) | -0.6 | 0.47 | 0.043 | 0.83 | -0.49 | -0.3 | 0.31 | -0.19 | 0.53 | 1 | -0.035 | 0.09 | 0.96 | -0.4 | -0.39 | -0.52 | 0.41 | 0.54 |  |
| Co# | 0.26 | -0.5 | -0.41 | -0.12 | 0.86 | 0.044 | 0.3 | -0.85 | 0.54 | -0.035 | 1 | 0.81 | 0.23 | 0.46 | 0.45 | 0.033 | -0.46 | 0.68 |  |
| Fe# | -0.15 | -0.3 | 0.15 | -0.018 | 0.67 | -0.44 | 0.67 | -0.53 | 0.28 | 0.093 | 0.81 | 1 | 0.32 | 0.64 | 0.57 | 0.35 | -0.67 | 0.6 |  |
| S# | -0.52 | 0.33 | -0.046 | 0.77 | -0.24 | -0.3 | 0.41 | -0.4 | 0.65 | 0.96 | 0.23 | 0.32 | 1 | -0.25 | -0.24 | -0.48 | 0.25 | 0.72 |  |
| As Val | 0.37 | -0.72 | 0.4 | -0.65 | 0.71 | -0.098 | 0.71 | 0.065 | 0.093 | -0.4 | 0.46 | 0.64 | -0.25 | 1 | 0.99 | 0.57 | -0.99 | 0.083 |  |
| As^5+^ | 0.48 | -0.81 | 0.35 | -0.71 | 0.7 | 0.051 | 0.68 | 0.054 | 0.2 | -0.39 | 0.45 | 0.57 | -0.24 | 0.99 | 1 | 0.44 | -0.96 | 0.054 |  |
| As^3+^ | -0.16 | 0.019 | 0.41 | -0.28 | 0.35 | -0.61 | 0.37 | 0.36 | -0.64 | -0.52 | 0.033 | 0.35 | -0.48 | 0.57 | 0.44 | 1 | -0.65 | -0.065 |  |
| As^1-^ | -0.29 | 0.65 | -0.42 | 0.61 | -0.71 | 0.19 | -0.72 | -0.069 | -0.019 | 0.41 | -0.46 | -0.67 | 0.25 | -0.99 | -0.96 | -0.65 | 1 | -0.1 |  |
| AVR | -0.22 | 0.074 | -0.41 | 0.53 | 0.37 | -0.32 | 0.44 | -0.65 | 0.63 | 0.54 | 0.68 | 0.6 | 0.72 | 0.083 | 0.054 | -0.065 | -0.1 | 1 |  |

| Supplementary Table I3. A correlation matrix showing the correlations of environmental and geochemical variables to each other for material from site C. | | | | | | | | | | | | | | | | | | | |
| --- | --- | --- | --- | --- | --- | --- | --- | --- | --- | --- | --- | --- | --- | --- | --- | --- | --- | --- | --- |
| **SITE C** | pH | Depth | Eh (V) | Ag (ppm) | As (wt%) | Ca (wt%) | Co (wt%) | Fe (wt%) | CO_2(T)_ (wt%) | S_(T)_ (wt%) | Co# | Fe# | S# | As Val | As^5+^ | As^3+^ | As^1-^ | AVR |  |
| pH | 1 | 0.74 | -0.27 | 0.27 | 0.72 | 0.23 | 0.39 | 0.65 | -0.89 | 0.34 | 0.63 | 0.78 | 0.34 | -0.25 | -0.38 | 0.75 | 0.22 | -0.13 |  |
| Depth | 0.74 | 1 | -0.76 | 0.57 | 0.97 | 0.53 | 0.83 | 0.16 | -0.66 | 0.84 | 0.98 | 0.39 | 0.84 | -0.71 | -0.8 | 0.9 | 0.69 | -0.29 |  |
| Eh (V) | -0.27 | -0.76 | 1 | -0.86 | -0.74 | -0.9 | -0.99 | 0.18 | 0.41 | -0.97 | -0.86 | 0.18 | -0.95 | 0.98 | 0.99 | -0.62 | -0.98 | 0.42 |  |
| Ag (ppm) | 0.27 | 0.57 | -0.86 | 1 | 0.65 | 0.9 | 0.82 | -0.16 | -0.36 | 0.74 | 0.71 | -0.34 | 0.71 | -0.86 | -0.89 | 0.64 | 0.85 | -0.66 |  |
| As (wt%) | 0.72 | 0.97 | -0.74 | 0.65 | 1 | 0.51 | 0.79 | 0.19 | -0.61 | 0.77 | 0.97 | 0.32 | 0.77 | -0.66 | -0.79 | 0.97 | 0.64 | -0.32 |  |
| Ca (wt%) | 0.23 | 0.53 | -0.9 | 0.9 | 0.51 | 1 | 0.88 | -0.12 | -0.47 | 0.82 | 0.65 | -0.26 | 0.79 | -0.94 | -0.91 | 0.42 | 0.94 | -0.5 |  |
| Co (wt%) | 0.39 | 0.83 | -0.99 | 0.82 | 0.79 | 0.88 | 1 | -0.11 | -0.51 | 0.99 | 0.91 | -0.05 | 0.98 | -0.98 | -0.99 | 0.67 | 0.97 | -0.42 |  |
| Fe (wt%) | 0.65 | 0.16 | 0.18 | -0.16 | 0.19 | -0.12 | -0.11 | 1 | -0.74 | -0.18 | 0.041 | 0.83 | -0.22 | 0.23 | 0.1 | 0.17 | -0.28 | 0.54 |  |
| CO_2(T)_ (wt%) | -0.89 | -0.66 | 0.41 | -0.36 | -0.61 | -0.47 | -0.51 | -0.74 | 1 | -0.45 | -0.59 | -0.71 | -0.42 | 0.41 | 0.5 | -0.56 | -0.37 | -0.036 |  |
| S_(T)_ (wt%) | 0.34 | 0.84 | -0.97 | 0.74 | 0.77 | 0.82 | 0.99 | -0.18 | -0.45 | 1 | 0.9 | -0.05 | 1 | -0.96 | -0.96 | 0.64 | 0.95 | -0.38 |  |
| Co# | 0.63 | 0.98 | -0.86 | 0.71 | 0.97 | 0.65 | 0.91 | 0.041 | -0.59 | 0.9 | 1 | 0.21 | 0.9 | -0.81 | -0.89 | 0.9 | 0.8 | -0.39 |  |
| Fe# | 0.78 | 0.39 | 0.18 | -0.34 | 0.32 | -0.26 | -0.05 | 0.83 | -0.71 | -0.05 | 0.21 | 1 | -0.05 | 0.22 | 0.11 | 0.3 | -0.25 | 0.45 |  |
| S# | 0.34 | 0.84 | -0.95 | 0.71 | 0.77 | 0.79 | 0.98 | -0.22 | -0.42 | 1 | 0.9 | -0.05 | 1 | -0.95 | -0.95 | 0.64 | 0.95 | -0.4 |  |
| As Val | -0.25 | -0.71 | 0.98 | -0.86 | -0.66 | -0.94 | -0.98 | 0.23 | 0.41 | -0.96 | -0.81 | 0.22 | -0.95 | 1 | 0.98 | -0.55 | -1 | 0.5 |  |
| As^5+^ | -0.38 | -0.8 | 0.99 | -0.89 | -0.79 | -0.91 | -0.99 | 0.1 | 0.5 | -0.96 | -0.89 | 0.11 | -0.95 | 0.98 | 1 | -0.69 | -0.97 | 0.46 |  |
| As^3+^ | 0.75 | 0.9 | -0.62 | 0.64 | 0.97 | 0.42 | 0.67 | 0.17 | -0.56 | 0.64 | 0.9 | 0.3 | 0.64 | -0.55 | -0.69 | 1 | 0.53 | -0.44 |  |
| As^1-^ | 0.22 | 0.69 | -0.98 | 0.85 | 0.64 | 0.94 | 0.97 | -0.28 | -0.37 | 0.95 | 0.8 | -0.25 | 0.95 | -1 | -0.97 | 0.53 | 1 | -0.53 |  |
| AVR | -0.13 | -0.29 | 0.42 | -0.66 | -0.32 | -0.5 | -0.42 | 0.54 | -0.04 | -0.38 | -0.39 | 0.45 | -0.4 | 0.5 | 0.46 | -0.44 | -0.53 | 1 |  |

# Supplementary Material Appendix J: Output Tables from NMDS Plots

| Supplementary Table J1. Output table from the NMDS plot in Figure 11, showing which variables are most significant in controlling the microbial community composition. The square brackets indicate concentration of the particular element or compound. An R^2^ and Pr (or p) value above 0.65 and less than 0.05 (respectively) is significant. | | | | |
| --- | --- | --- | --- | --- |
| **SITES A - C** | **NMDS1** | **NMDS2** | **R^2^** | **Pr(>r)** |
| pH | -0.608 | -0.794 | 0.333 | 0.050 (.) |
| Depth | 0.287 | 0.958 | 0.540 | 0.005 (**) |
| Eh | -0.023 | 1.000 | 0.284 | 0.081 (.) |
| [Ag] | 0.493 | -0.870 | 0.544 | 0.004 (**) |
| [As] | 0.325 | 0.946 | 0.404 | 0.019 (*) |
| [Ca] | -0.287 | 0.958 | 0.335 | 0.038 (*) |
| [Co] | 0.288 | 0.958 | 0.308 | 0.053 (.) |
| [Fe] | 0.082 | 0.997 | 0.236 | 0.116 |
| [CO_2(T)_] | 0.934 | 0.356 | 0.196 | 0.185 |
| [S_(T)_] | 0.251 | 0.968 | 0.289 | 0.074 (.) |
| Co# | 0.532 | 0.846 | 0.406 | 0.022 (*) |
| Fe# | -0.874 | 0.485 | 0.737 | 0.001 (***) |
| S# | -0.080 | 0.997 | 0.183 | 0.227 |
| Significant codes: 0 (***), 0.001 (**), 0.01 (*), 0.05 (.), 0.1 ( ) | | | | |

| Supplementary Table J2. Output table from the NMDS plot in Figure 12a, showing which variables are most significant in controlling the microbial community composition in site A. The square brackets indicate concentration of the particular element or compound. An R^2^ and Pr (or p) value above 0.65 and less than 0.05 (respectively) is significant. | | | | |
| --- | --- | --- | --- | --- |
| **SITE A** | **NMDS1** | **NMDS2** | **R^2^** | **Pr(>r)** |
| pH | -0.904 | -0.428 | 0.231 | 0.644 |
| Depth | 0.799 | 0.601 | 0.961 | 0.011 (*) |
| Eh | 0.974 | 0.226 | 0.034 | 0.956 |
| [Ag] | 0.598 | -0.802 | 0.882 | 0.047 (*) |
| [As] | -0.804 | -0.595 | 0.200 | 0.697 |
| [Ca] | -0.853 | -0.552 | 0.083 | 0.875 |
| [Co] | -0.946 | 0.324 | 0.422 | 0.442 |
| [Fe] | -0.987 | 0.158 | 0.193 | 0.779 |
| [CO_2(T)_] | 0.471 | -0.882 | 0.249 | 0.661 |
| [S_(T)_] | -0.984 | 0.177 | 0.767 | 0.081 (.) |
| Co# | -0.903 | 0.429 | 0.293 | 0.657 |
| Fe# | -0.484 | 0.875 | 0.309 | 0.611 |
| S# | -0.441 | 0.897 | 0.510 | 0.349 |
| Significant codes: 0.01 (**), 0.01 (*), 0.05 (.), 0.1 ( ) | | | | |

| Supplementary Table J3. Output table from the NMDS plot in Figure 12b, showing which variables are most significant in controlling the microbial community composition in site B. The square brackets indicate concentration of the particular element or compound. AVR (average valence ratio) is the ratio of the average positive versus negative arsenic species (Equation 5). An R2 and Pr (or p) value above 0.65 and less than 0.05 (respectively) is significant. Significant codes: 0.01 (**), 0.01 (*), 0.05 (.), 0.1 ( ) | | | | |
| --- | --- | --- | --- | --- |
| **SITE B** | **NMDS1** | **NMDS2** | **R^2^** | **Pr(>r)** |
| pH | -0.513 | 0.859 | 0.915 | 0.031 (*) |
| Depth | 0.855 | -0.519 | 0.936 | 0.005 (**) |
| Eh | -0.260 | -0.966 | 0.919 | 0.007 (**) |
| [Ag] | 0.902 | -0.431 | 0.579 | 0.225 |
| [As] | -0.682 | 0.731 | 0.352 | 0.497 |
| [Ca] | -0.313 | 0.949 | 0.775 | 0.087 (.) |
| [Co] | -0.563 | -0.826 | 0.643 | 0.221 |
| [Fe] | -0.142 | -0.989 | 0.243 | 0.651 |
| Co# | -0.543 | 0.840 | 0.180 | 0.724 |
| Fe# | -0.612 | -0.791 | 0.187 | 0.717 |
| S# | 0.433 | -0.901 | 0.067 | 0.936 |
| [CO_2(T)_] | -0.577 | 0.817 | 0.326 | 0.571 |
| [S_(T)_] | -0.513 | -0.858 | 0.1352 | 0.779 |
| Average As valence | -0.937 | -0.350 | 0.703 | 0.171 |
| As^5+^ | -0.982 | -0.186 | 0.811 | 0.118 |
| As^3+^ | -0.061 | -0.998 | 0.208 | 0.765 |
| As^1-^ | 0.889 | 0.458 | 0.642 | 0.178 |
| AVR | 0.58110 | 0.81383 | 0.0219 | 0.975 |

| Supplementary Table J4. Output table from the NMDS plot in Figure 12c, showing which variables are most significant in controlling the microbial community composition in site C. The square brackets indicate concentration of the particular element or compound. AVR (average valence ratio) is the ratio of the average positive versus negative arsenic species (Equation 5). An R^2^ and Pr (or p) value above 0.65 and less than 0.05 (respectively) is significant. Significant codes: 0.01 (**), 0.01 (*), 0.05 (.), 0.1 ( ) | | | | |
| --- | --- | --- | --- | --- |
| **SITE C** | **NMDS1** | **NMDS2** | **R^2^** | **Pr(>r)** |
| pH | 0.769 | -0.639 | 0.786 | 0.048 (*) |
| Depth | 0.956 | 0.293 | 0.933 | 0.0278 (*) |
| Eh | -0.442 | -0.897 | 0.833 | 0.0167 (*) |
| [Ag] | 0.380 | 0.925 | 0.539 | 0.319 |
| [As] | 0.937 | 0.349 | 0.956 | 0.030 (*) |
| [Ca] | 0.255 | 0.967 | 0.482 | 0.400 |
| [Co] | 0.557 | 0.831 | 0.770 | 0.046 (*) |
| [Fe] | 0.30421 | -0.953 | 0.325 | 0.558 |
| Co# | 0.852 | 0.524 | 0.925 | 0.026 (*) |
| Fe# | 0.446 | -0.895 | 0.719 | 0.126 |
| S# | 0.576 | 0.817 | 0.782 | 0.054 (.) |
| [CO_2(T)_] | -0.878 | 0.477 | 0.385 | 0.558 |
| [S_(T)_] | 0.560 | 0.829 | 0.791 | 0.042 (*) |
| Average As valence | -0.404 | -0.915 | 0.690 | 0.090 (.) |
| As^5+^ | -0.523 | -0.853 | 0.767 | 0.061 (.) |
| As^3+^ | 0.986 | 0.165 | 0.874 | 0.061 (.) |
| As^1-^ | 0.38384 | 0.9234 | 0.687 | 0.093 (.) |
| AVR | -0.544 | -0.839 | 0.075 | 0.844 |

# Supplementary Material Appendix K: Silver Concentration versus Depth

|  |
| --- |
| Supplementary Figure K1. Depth profile plots showing the variation in Ag concentrations with depth (cm). |
